# *NRN1* epistasis with *BDNF* and *CACNA1C*: mediation effects on symptom severity through neuroanatomical changes in schizophrenia

**DOI:** 10.1007/s00429-024-02793-5

**Published:** 2024-05-09

**Authors:** Carmen Almodóvar-Payá, Maria Guardiola-Ripoll, Maria Giralt-López, Maitane Oscoz-Irurozqui, Erick Jorge Canales-Rodríguez, Mercè Madre, Joan Soler-Vidal, Núria Ramiro, Luis F. Callado, Bárbara Arias, Carme Gallego, Edith Pomarol-Clotet, Mar Fatjó-Vilas

**Affiliations:** 1 FIDMAG Germanes Hospitalàries Research Foundation, Barcelona, Spain; 2 Departament de Biologia Evolutiva, Ecologia i Ciències Ambientals, Facultat de Biologia, Universitat de Barcelona, Barcelona, Spain; 3 CIBERSAM (Biomedical Research Network in Mental Health), Instituto de Salud Carlos III, Madrid, Spain; 4 Department of Child and Adolescent Psychiatry, Germans Trias i Pujol University Hospital (HUGTP), Barcelona, Spain; 5 Department of Psychiatry and Legal Medicine, Faculty of Medicine, Autonomous University of Barcelona (UAB), Barcelona, Spain; 6 Red de Salud Mental de Gipuzkoa, Osakidetza-Basque Health Service, Gipuzkoa, Spain; 7 Signal Processing Laboratory (LTS5), École Polytechnique Fédérale de Lausanne (EPFL), Lausanne, Switzerland; 8 Mental Health, IR SANT PAU, Hospital de la Santa Creu i Sant Pau, Universitat Autònoma Barcelona, Barcelona, Spain; 9 Hospital Benito Menni, Germanes Hospitalàries, Sant Boi de Llobregat, Barcelona, Spain; 10 Hospital San Rafael, Germanes Hospitalàries, Barcelona, Spain; 11 Department of Pharmacology, University of the Basque Country (UPV/EHU), Bizkaia, Spain; 12 BioBizkaia Health Research Institute, Bizkaia, Spain; 13 Institut de Biomedicina de la Universitat de Barcelona (IBUB), Barcelona, Spain; 14 Department of Cells and Tissues, Molecular Biology Institute of Barcelona (IBMB-CSIC), Barcelona, Spain; 15 CIBERER (Biomedical Research Network in Rare Diseases), Instituto de Salud Carlos III, Madrid, Spain

**Keywords:** Schizophrenia, Genetic epistasis, Symptoms, Neuroimaging, Mediation

## Abstract

The expression of Neuritin-1 (*NRN1*), a neurotrophic factor crucial for neurodevelopment and synaptic plasticity, is enhanced by the Brain Derived Neurotrophic Factor (*BDNF*). Although the receptor of NRN1 remains unclear, it is suggested that NRN1’s activation of the insulin receptor (IR) pathway promotes the transcription of the calcium voltage-gated channel subunit alpha1 C (*CACNA1C*). These three genes have been independently associated with schizophrenia (SZ) risk, symptomatology, and brain differences. However, research on how they synergistically modulate these phenotypes is scarce. We aimed to study whether the genetic epistasis between these genes affects the risk and clinical presentation of the disorder via its effect on brain structure. First, we tested the epistatic effect of *NRN1* and *BDNF* or *CACNA1C* on (i) the risk for SZ, (ii) clinical symptoms severity and functionality (onset, PANSS, CGI and GAF), and (iii) brain cortical structure (thickness, surface area and volume measures estimated using FreeSurfer) in a sample of 86 SZ patients and 89 healthy subjects. Second, we explored whether those brain clusters influenced by epistatic effects mediate the clinical profiles. Although we did not find a direct epistatic impact on the risk, our data unveiled significant effects on the disorder’s clinical presentation. Specifically, the *NRN1*-rs10484320 x *BDNF*-rs6265 interplay influenced PANSS general psychopathology, and the *NRN1*-rs4960155 x *CACNA1C*-rs1006737 interaction affected GAF scores. Moreover, several interactions between *NRN1* SNPs and *BDNF*-rs6265 significantly influenced the surface area and cortical volume of the frontal, parietal, and temporal brain regions within patients. The *NRN1*-rs10484320 x *BDNF*-rs6265 epistasis in the left lateral orbitofrontal cortex fully mediated the effect on PANSS general psychopathology. Our study not only adds clinical significance to the well-described molecular relationship between *NRN1* and *BDNF* but also underscores the utility of deconstructing SZ into biologically validated brain-imaging markers to explore their mediation role in the path from genetics to complex clinical manifestation.

**Supplementary Information:**

The online version contains supplementary material available at 10.1007/s00429-024-02793-5.

## Background

Schizophrenia (SZ) is a severe psychiatric disorder with a substantial burden affecting 21 million people worldwide (Charlson et al. 2018). It has a strong genetic component that is reflected in its high heritability (*h*^*2*^ = 65–79%) (Hilker et al. 2018; Sullivan et al. 2003). Moreover, genome-wide association studies (GWAS) have provided solid evidence of its polygenicity by describing a hundred genetic variants with additive effects (Pardiñas et al. 2018; Trubetskoy et al. 2022). Interestingly, due to the convergence of those variants in molecular networks related to synaptic plasticity, it has been proposed as a key pathophysiological mechanism in SZ (Hall and Bray 2022). However, the identified risk variants together only account for a proportion (*h*^*2*^_*SNP*_ = 24%) of the variance in liability of the phenotype (Trubetskoy et al. 2022).

It has been suggested that such *missing heritability* could arise, among other factors, from nonlinear molecular interactions, which are not typically explored in classical GWAS models (Zuk et al. 2012). In fact, gene-gene interactions between two or more loci have been extensively studied in animal and cellular models, suggesting that epistatic networks might represent an essential molecular mechanism involved in the modulation of complex traits (Mackay and Moore 2014; Özsoy et al. 2021). When the collective effect of large-scale genetic interactions is considered in humans, the association with complex traits, such as SZ, becomes more robust and new variants appear (Woo et al. 2017). Therefore, considering that genetic interactions mainly occur between genes involved in the same molecular pathways (Roguev et al. 2008), inspecting epistatic effects among those involved in synaptic plasticity in SZ may add relevant data on how genetic factors lead to the emergence of the disorder.

Considering the phenotypic complexity of SZ as reflected in its diverse outcomes, neuroimaging genetics approaches provide a neurobiological context for studying how genetic variants act to confer an increased risk for the disorder (van der Meer and Kaufmann 2022). Notably, SZ-related genes identified by GWAS are highly expressed in brain regions with structural differences in SZ (Ji et al. 2021) and have been associated with symptomatology (Legge et al. 2021; Sengupta et al. 2017). This indicates a partial genetic overlap within the axis connecting the brain and symptoms, with specific genes delineated as potential contributors to cerebral alterations that sustain distinct manifestations. Indeed, some studies have shown that genetic effects on clinical phenotypes are mediated by specific brain regions and functions in mental disorders (Sudre et al. 2020). Nevertheless, few cases in the literature explore the impact of epistasis on brain phenotypes (Callicott et al. 2013; Guardiola-Ripoll et al. 2022; Tecelão et al. 2019; Xu et al. 2018) and its mediation effect on clinical symptoms in SZ. These studies have highlighted the methodological challenges in exploring epistasis while effectively unveiling non-independent effects between those genes that go unnoticed when examining main effects alone. Therefore, such approaches reveal the important role of gene interactions in the architecture of common human diseases while helping to connect the statistical perspective with the complex dynamics of biological systems (Phillips 2008).

One of the genes intimately implicated in synaptic plasticity processes is Neuritin-1 (*NRN1*, 6p25.1), which encodes for a neurotrophin that is highly expressed in the hippocampus, the cerebral cortex and the cerebellum (Naeve et al. 1997). Its expression is experience-dependent (Harwell et al. 2005; Nedivi et al. 1996) and regulated by Ca^2+^ influx via the N-methyl-D-aspartate (NMDA) receptors (Fujino et al. 2003). Additionally, it has been described that another neuropeptide, the Brain-Derived Neurotrophic Factor (*BDNF*, 11p13), modulates *NRN1* expression. This gene is highly expressed in the same cerebral regions as *NRN1* (Esvald et al. 2023) and also in an experience-dependent manner (Tongiorgi 2008). As neurotrophins, both genes exert multiple functions in the developing brain, being involved, for example, in enhancing neurite and dendritic growth, stabilising active synapses, improving synaptic maturation, increasing neuronal migration and regulating apoptosis of proliferative neurons, but are also involved in regulating the neuronal plasticity in the adult brain (Sasi et al. 2017; Yao et al. 2018). In the synaptic cleft, BDNF binds tyrosine kinase receptor B (TrkB) activating the transcription factor CREB (cAMP response element-binding protein) that attaches, among others, to the endogenous *NRN1* promoter in vivo (Finkbeiner et al. 1997; Fujino et al. 2003) fostering functional and structural neuronal changes and leading to the consolidation of long-term synaptic plasticity (Kaldun and Sprecher 2019). Besides, animal-based models have described the direct relationship between *BDNF* and *NRN1* expression, showing that the intraventricular injection or the intrahippocampal infusion of BDNF into neonatal rat pups results in the up-regulation of *NRN1* expression in vivo (Wibrand et al. 2006).

Current research looking for potential receptors for *NRN1* suggests a role in regulating synaptic excitability through the activation of the insulin receptor (IR) and downstream pathway, which trigger the transcription and trafficking of L-type voltage-gated calcium channel (L-VGCC) subunits to the membrane of cortical neurons, specifically Cav1.3 and Cav1.2 (Lu et al. 2017; J.-J. Yao et al. 2012; Zhao et al. 2018). The latter is encoded by humans’ calcium voltage-gated channel subunit alpha1 C gene (*CACNA1C*, 12p13.33). Notably, L-VGCC channels constitute the most abundant calcium channels in the human brain, accounting for approximately 90% (Striessnig et al. 2014). These channels are located at the post-synapsis, specifically at the neurons’ soma and dendritic spines and shaft (Jenkins et al. 2010). There, they facilitate Ca^2+^ influx in response to membrane depolarisation, which acts as a cellular messenger that triggers diverse cellular responses, including the strengthening of short- and long-term synaptic plasticity and the promotion of activity-dependent gene expression in a process called excitation-transcription coupling (Ma et al. 2011).

The genetic association of *BDNF* and *CACNA1C* genes has been widely reported in psychiatric disorders, and *NRN1*, while far less explored, has been identified as an interesting candidate gene, involved in age at onset, general cognitive abilities and brain activity in SZ (Almodóvar-Payá et al. 2022; Chandler et al. 2010; Fatjó-Vilas et al. 2016). Regarding the role of *BDNF* in SZ, the rs6265 polymorphism has been associated not only with the risk for the disorder (Kheirollahi et al. 2016; Rosa et al. 2006) but also with a range of clinical features, including the age of onset, symptoms, therapeutic responsiveness, neurocognitive function and brain morphology and activity (see review by Notaras et al. 2005). About *CACNA1C* variability, the rs1006737 polymorphism has been repeatedly associated with the risk for SZ both through GWAS and meta-analysis (Trubetskoy et al. 2022; Zhu et al. 2019) and with disrupted cognitive performance (Novaes de Oliveira Roldan et al., 2023) and altered brain structure and function in subjects with SZ (Gurung and Prata 2015). However, whether epistatic effects among these genes are involved in the aetiology of SZ remains largely unexplored. Two preceding studies have suggested that *NRN1* x *BDNF*-rs6265 epistasis is associated with the susceptibility for SZ-spectrum disorders and modulates depressive symptoms in healthy subjects (HS) (Fatjó-Vilas et al. 2016; Prats et al. 2017). In summary, the currently available molecular and genetic association data point towards the potential synergistic role of *NRN1, BDNF* and *CACNA1C* in the risk of SZ and the modulation of the disorder’s presentation.

Consequently, we hypothesised that genetic epistasis between *NRN1, BDNF* and *CACNA1C* would be associated with the clinical manifestations of the disorder and that such effects would be mediated by their modulation of brain cortical structure. To test this hypothesis, first, we explored the impact of polymorphic variability along the *NRN1* gene in combination with *BDNF* (rs6265) or *CACNA1C* (rs1006737) on (i) the risk for SZ; (ii) the clinical severity and functionality plus age at onset; and, (iii) the neuroanatomical cortical measures (thickness (CT), surface area (CSA) and volume (CV)). Second, we investigated whether those brain clusters under a significant epistatic effect might be mediating the clinical outcomes of the patients.

## Methods

### Sample

The sample consisted of a case-control dataset of 175 individuals: 86 healthy subjects (HS) and 89 subjects diagnosed with SZ. All participants provided a biological sample for genotypic analyses and underwent a comprehensive clinical evaluation and a magnetic resonance imaging (MRI) session, detailed in the subsequent sections. The subjects with SZ were recruited from Germanes Hospitalaries psychiatric hospitals in the Barcelona area (Hospital Benito Menni and Hospital Sant Rafael), and the healthy controls were recruited from the same area.

All participants were of European origin with ages between 18 and 65 years, had an Intelligence Quotient (IQ) > 75 according to the Wechsler Adult Intelligence Scale III (WAIS-III) (Wechsler 1997) and were right-handed. Experienced psychiatrists evaluated patients using the Structured Clinical Interview for DSM Disorders (SCID) (First et al. 2002) and met the DSM-IV-TR criteria for SZ. The HS had no personal history of mental disorders or treatment. All participants met the same exclusion criteria, which included a major medical illness affecting brain function, neurological conditions, a history of head trauma with loss of consciousness and a history of drug abuse or dependence.

#### Ethical approval

was obtained from the research ethics committee, and all participants provided written consent after being informed about the study procedures and implications. All procedures were carried out according to the Declaration of Helsinki.

### Clinical assessment

The patients with SZ underwent a clinical evaluation including (i) the age at onset of the first episode, based on the first appearance of psychotic symptoms and established by experienced psychiatrists through clinical information derived from case notes and information provided by the patient and close relatives; (ii) symptom severity, evaluated with the Positive and Negative Symptoms Scale (PANSS) (Kay et al. 1987); (iii) the Global Assessment of Functioning (GAF) (Endicott et al. 1976); and, (iv) the Clinical Global Impression scale (CGI) (Table 1).

**Table 1:** Sample characteristics, including a demographic and clinical description of the healthy subjects and the subjects diagnosed with schizophrenia (SZ) of the study

	Healthy subjects (*n* = 86)	Subjects with SZ (*n* = 89)	
Sex (females / males)	49/37 (57)	26/63 (29)	X^2^ = 13.78; *p* < 0.001
Age at interview	38.57 (11.02)	39.47 (10.88)	t=-0.55; *p* = 0.587
Illness duration ^a^	-	16.67 (11.01)	-
CPZ equivalents ^b^	-	630 (504.91)	-
ICV	1517379.54 (152541.18)	1554805.55 (146504.42)	t=-1.656; *p* = 0.100
Age of onset ^a^	-	22.60 (6.38)	-
PANSS Positive	-	17.34 (5.95)	-
PANSS Negative	-	20.47 (7.68)	-
PANSS General Psychopathology	-	33.56 (9.77)	-
PANSS Total	-	71.37 (19.66)	-
GAF	-	46.33 (15.13)	-
CGI	-	4.54 (1.05)	-

Mean and standard deviation (sd) are reported for the quantitative variables, while count and percentage (%) are given for the qualitative variables. For patients, illness duration is shown in years, and chlorpromazine (CPZ) equivalents are in mg/day. Regarding clinical evaluation, the age at onset in years, the subscales of the Positive and Negative Symptoms Scale (PANSS), the Global Assessment of Functioning (GAF) scale and the Clinical Global Impression (CGI) scale are reported. Concerning neuroimaging assessment, intracranial volume (ICV) is given in mm^3^ for patients and controls

^a^ Data of age at onset was available for 86 subjects with SZ. Illness duration was quantified as the patient’s chronological age minus the age of onset

^b^ Data of CPZ equivalent was available for 85 subjects with SZ

### Genotyping

Genomic DNA was obtained for all individuals from buccal mucosa through cotton swabs and extracted using ATP Genomic DNA Mini Kit Tissue (Teknokroma Analítica, S.A., Sant Cugat del Vallès, Barcelona, Spain) or peripheral blood cells by punction and extracted using Realpure SSS Kit for DNA Extraction (Durviz, S.L.U, Valencia, Spain). We genotyped: (i) eleven single nucleotide polymorphisms (SNP) at the Neuritin1 gene (*NRN1*, 6p25.1), (ii) the SNP rs6265 (also known as Val66Met; GRCh38 position: 27,658,369) at the Brain-Derived Neurotrophic Factor gene (*BDNF*, 11p13) for which the T allele encodes for the amino acid methionine (Met) and the C allele encodes for valine (Val), and (iii) the SNP rs1006737 (GRCh38 position: 2,236,129) at calcium voltage-gated channel subunit alpha1 gene (*CACNA1C*, 12p13.33). The genotyping was carried out at the National Genotyping Center (CeGen) of the Network Platform of Biomolecular and Bioinformatics Resources (PRB3) of the Carlos III Health Institute (ISCIII). The genotyping call rate was 97% and, as shown in Table 2, the minor allele frequencies were comparable to the ones described for the EUR population in the 1,000 Genomes Project, and the genotype frequencies were in Hardy-Weinberg equilibrium (PLINK v.1.07) (Purcell et al. 2007).

**Table 2:** Information on *NRN1*, *BDNF* and *CACNA1C* SNPs that were included in this study

Gene	SNPs	Allele^a^	MAF_1000G_	MAF_sample_	HS genotypic counts (%)^b^			SZ genotypic counts (%)^b^		
*NRN1*	rs2208870	G/A	0.34	0.33	7(0.08)	43(0.5)	36(0.42)	10(0.11)	42(0.47)	37(0.42)
	rs12333117	T/C	0.40	0.40	15(0.18)	34(0.4)	36(0.42)	16(0.18)	42(0.47)	31(0.35)
	rs582186	A/G	0.38	0.39	12(0.14)	41(0.48)	32(0.38)	15(0.17)	43(0.49)	30(0.34)
	rs645649	C/G	0.36	0.38	10(0.12)	47(0.55)	29(0.34)	11(0.12)	49(0.55)	29(0.33)
	rs582262	C/G	0.30	0.28	9(0.1)	35(0.41)	42(0.49)	6(0.07)	35(0.4)	46(0.53)
	rs3763180	T/G	0.46	0.46	25(0.29)	42(0.49)	18(0.21)	14(0.16)	36(0.4)	39(0.44)
	rs10484320	T/C	0.22	0.22	5(0.06)	23(0.27)	58(0.67)	9(0.1)	33(0.37)	47(0.53)
	rs4960155	C/T	0.49	0.52	26(0.33)	40(0.5)	14(0.18)	19(0.21)	40(0.45)	30(0.34)
	rs9379002	G/T	0.29	0.26	6(0.07)	28(0.33)	51(0.6)	7(0.08)	35(0.4)	46(0.52)
	rs9405890	C/T	0.31	0.33	8(0.09)	33(0.38)	45(0.52)	14(0.16)	39(0.44)	36(0.4)
	rs1475157	G/A	0.17	0.15	4(0.05)	15(0.17)	67(0.78)	3(0.03)	23(0.26)	63(0.71)
*BDNF*	rs6265	T/C	0.20	0.21	2(0.02)	27(0.31)	57(0.66)	5(0.06)	34(0.38)	50(0.56)
*CACNA1C*	rs1006737	A/G	0.32	0.29	11(0.13)	29(0.34)	46(0.53)	9(0.1)	33(0.37)	47(0.53)

The table reports on the dbSNP number, the alleles, and the minor allele frequency (MAF) for each SNP according to 1000 Genomes Project (1000G) Phase 3 in European (EUR) population. The MAF and the genotypic count (frequencies) observed in the study sample are also given for each SNP

^a^ The minor allele described in the 1000 Genomes Project for the EUR population is placed first

^b^ The homozygous for the minor allele according to the EUR population of the 1000 Genomes project is placed first, then the heterozygous, and last the homozygous for the major allele

### MRI data acquisition, processing, and analyses

The neuroimaging protocol was conducted at the Hospital Sant Joan de Déu using a 1.5T GE Sigma MRI scanner. High-resolution structural-T1 imaging was obtained using the following acquisition parameters: matrix size = 512 × 512; 180 contiguous axial slices; voxel-resolution 0.47 × 0.47 × 1 mm3; echo (TE), repetition (TR) and inversion (TI) times, (TE / TR / TI) = 3.93 / 2000 / 710 ms, respectively; flip angle of 15°. This scan was used to estimate the three cortical measures: cortical thickness (CT), cortical surface area (CSA) and cortical volume (CV).

Structural MRI data were analysed with the FreeSurfer (http://surfer.nmr.mgh.harvard.edu/). Briefly, the pre-processing included the removal of non-brain tissue, automated Talairach transformation, tessellation of the grey and white matter boundaries and surface deformation (Fischl et al. 2004), after which individual images were normalised to a common stereotaxic space. Some deformation procedures were performed in the data analysis pipeline, including surface inflation and registration to a spherical atlas. This method uses both intensity and continuity information from the entire three-dimensional images in the segmentation and deformation procedures to produce vertex-wise representations of CT, CSA, and CV.

All subjects included in this study passed the standardised quality-control protocols from the ENIGMA consortium (http://enigma.ini.usc.edu/protocols/imaging-protocols) that have previously been applied in large-scale multi-centre studies (Hibar et al. 2018).

### Statistical analyses

#### Epistasis models between *NRN1* and its interactors (*BDNF* and *CACNA1C)* on SZ phenotypes

Taking into account previously reported association data, and to maximise the power of the detected gene-gene interactions, all the analyses were carried out grouping the minor and the heterozygous genotypes for all the *NRN1* SNPs, for the *BDNF*-rs6265 (Met-allele carriers and Val/Val homozygotes) and *CACNA1C*-rs1006737 (A-allele carriers and GG homozygotes; Supplementary Table S1 and S2).

First, the epistatic effects on the risk for the disorder were studied using two-way interaction factors in logistic regression models (adjusted by sex). Second, we used linear regression models to test the gene-gene effects on the clinical data within subjects with SZ (adjusted by age and sex). We only considered those interactions significant after the Bonferroni correction (0.05 / 11 = 0.0045, based on the number of SNPs analysed). Statistical power calculations were performed using version 1.0.2 of the ‘genpwr’ R package, considering a minor allele frequency (MAF) range of 0.17 to 0.49 and a power of 0.80. In the case of epistasis analyses on the risk (case-control logistic regression), we had a detectable odds ratio ≥ 1.85. For the epistasis analyses on the clinical measures (linear regressions within patients), we had a potentially detectable regression coefficient ≥ 1.76. Third, the impact of genetic interactions on brain measurements was explored within subjects with SZ using the Qdec graphical user interface that implements the general linear models. Specifically, our epistasis analyses were based on the two-factor, two-level ANOVA implemented in the FreeSurfer software, with the two genes (*NRN1* and the interactor, *BDNF* or *CACNA1C*) as groups and three covariates (sex, age, and intracranial volume (ICV)). We used a Monte Carlo Null-Z simulation with a z-value threshold of 2.3 (equivalent to *p* < 0.005, two tails) to correct for multiple comparisons. In these analyses, according to the Bonferroni threshold mentioned before, only those clusters with cluster-wise p-threshold that met that criterion were considered. Anatomical locations of the significant regions were determined using the surface atlas included in the FreeSurfer software. Fourth, the values for each significant cluster were imported to SPSS to explore their association with clinical measures using linear regression models (adjusted by age and sex), for graphical purposes and further moderated mediation analyses.

#### Moderated mediation analyses

**Fig. 1:**
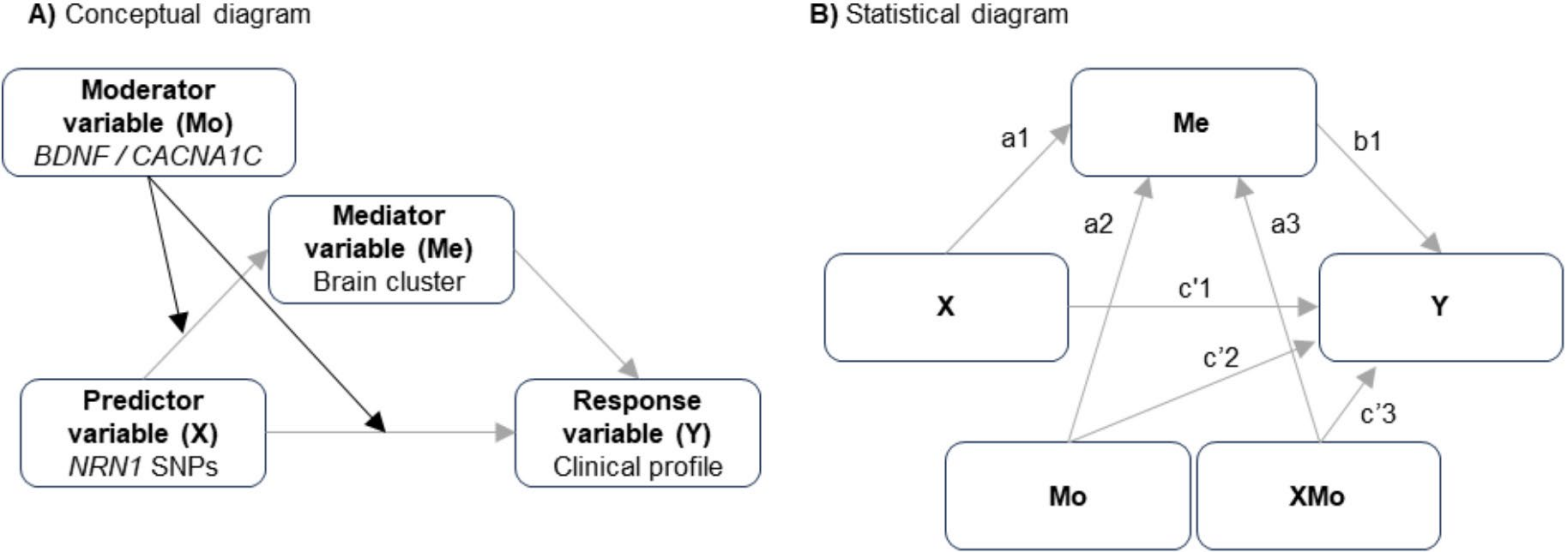
Schematic representation of the different moderated mediation models used. **(A)** Conceptual diagrams representing the used model. The grey arrows indicate the indirect effect of X on Y through Me and modulated by Mo. **(B)** Statistical diagram depicting the different equations used in the moderated mediation model. Two equations are used to estimate the index of moderation mediation (IMM): first, the effect of X (a1) and Mo (a2) variables and their interaction (a3) on Me (model 1: Me = β0 + β1X + β2Mo + β3XMo + ε); second, the effect of X (c’1), Mo (c’2), their interaction (c’3) and Me (b1) on Y (model 2: Y = β0 + β1Me + β2X + β3Mo + β4XMo + ε). The different letters represent the coefficients of each one of these effects. Then, the index of moderated mediation was calculated as the product of the coefficients a3*b1

A moderated mediation model included those epistatic models significantly associated with clinical profiles and neuroanatomical measures (Fig. 1). This approach allowed us to investigate whether those brain clusters where we detected significant epistasis between *NRN1* and *BDNF* or *CACNA1C* might mediate the effect detected on clinical profiles. We built a moderated mediation analysis with *NRN1* as the predictor variable (X), clinical features as the response variable (Y), *BDNF*-rs6265 or *CACNA1C*-rs1006737 as the moderator variable (Mo), and the brain measures as the mediating variable (Me). We used the R package “mediation” (Tingley et al. 2014). This tests, first, if the effect of X on the Me differs conditional to Mo (model 1: Me = β0 + β1X + β2Mo + β3XMo + ε), and second, if Me and the interaction between X and Mo are significantly associated with Y (model 2: Y = β0 + β1Me + β2X + β3Mo + β4XMo + ε). Lastly, it allows the calculation of the indirect effect (the index of moderation mediation), which is quantified as the product of the regression coefficient for the X and Mo interaction in model 1 and the regression coefficient of Me in model 2 (βindirect = β3XMo * β1Me). Once the indirect effect is calculated, the confidence interval and the significance levels for the entire indirect effect are estimated based on the bootstrap method with 10,000 resampling iterations. The conceptual and statistical diagrams of the model are shown in Fig. 1.

## Results

### Sample characteristics

Table 1 shows the main sociodemographic and clinical data of the sample. As shown, subjects with SZ and HS presented sex differences; then, this variable was used as a covariable in the analyses exploring the epistatic effect on the risk.

### Epistatic effects on the risk for SZ

There were no *NRN1* x *BDNF*-rs6265 nor *NRN1* x *CACNA1C*-rs1006737 epistatic effects on the risk for the disorder (Supplementary Table S1 and S1).

### Epistatic effects on SZ clinical measures

**Fig. 2:**
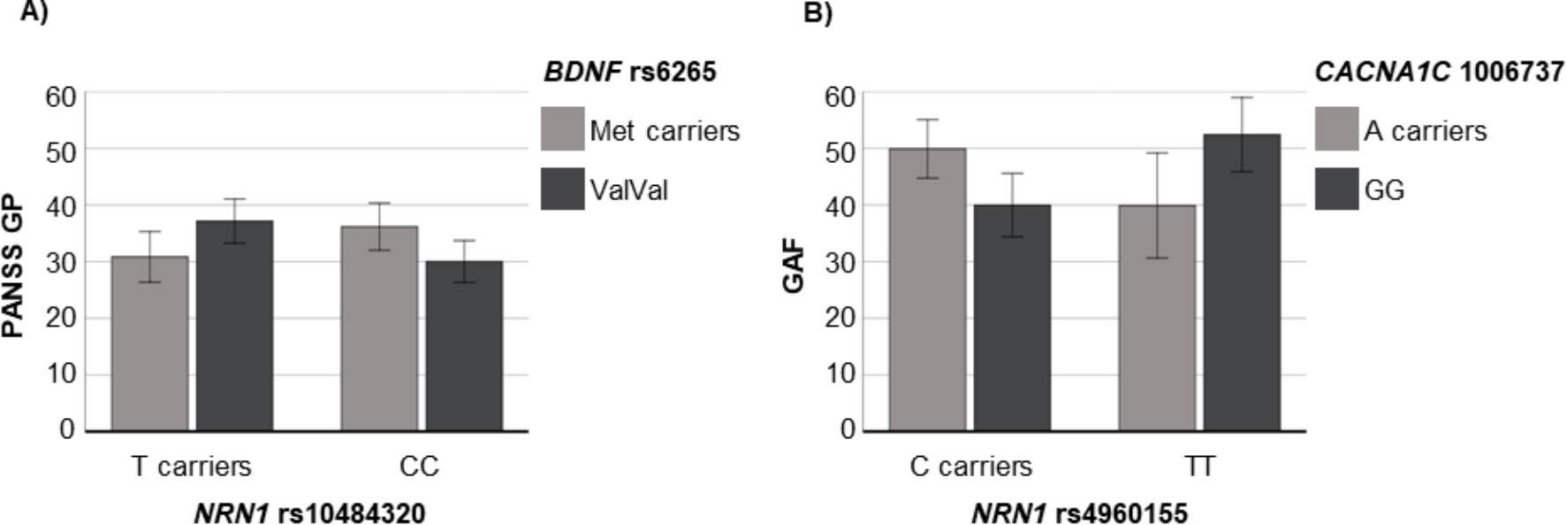
Bar plots showing the significant epistatic effects detected on clinical measures within SZ subjects. Each bar represents the scores’ marginal mean (± 2 standard error) for each epistatic group. **A**) *NRN1*-rs10484320 x *BDNF*-rs6265 interaction on Positive and Negative Symptoms Scale (PANSS) general psychopathology. **B**) *NRN1*-rs4960155 x *CACNA1C*-rs1006737 interaction on Global Assessment of Functioning (GAF)

We detected a significant two-order gene-gene interaction between *NRN1*-rs10484320 and *BDNF*-rs6265 on PANSS general psychopathology. As shown in Table 3, while the main effects of *NRN1-*rs10484320 and *BDNF*-rs6265 were not detected, the interaction of both was significant. Adding the interaction term significantly improved the model’s overall fit (Δ-R^2^ = 0.098, p-value of the change = 0.003). As illustrated in Fig. 2A, patients presented inverse patterns in PANSS general psychopathology scores as a function of both *NRN1* and *BDNF* genotypes. Remarkably, the significance of this effect is amplified with the inclusion of treatment, as chlorpromazine (CPZ) equivalents in mg/day, as a covariate in the model (β = -0.651, SE = 4.154, *p *= 0.001, model Adj-R^2^ = 0.099).

**Table 3:** Linear regression models showing significant *NRN1* epistasis with *BDNF* or *CACNA1C* on clinical measures

Clinical outcomes	Models	Genetic predictors	β	SE	p-value
Epistasis with *BDNF*					
PANSS General Psychopathology ^a^	i) Main effects	*NRN1*-rs10484320	-0.087	2.105	0.426
		*BDNF*-rs6265	-0.018	2.146	0.873
	ii) Interaction	*NRN1*-rs10484320 *x BDNF*-rs6265	-0.583	4.076	0.003
Epistasis with *CACNA1C*					
GAF ^b^	i) Main effects	*NRN1*-rs4960155	0.111	3.468	0.312
		*CACNA1C*-rs1006737	-0.100	3.284	0.346
	ii) Interaction	*NRN1*-rs4960155 x *CACNA1C*-rs1006737	0.233	6.727	0.002

Each factor effect is given (β, standardized regression coefficient; SE, standard error), and the global statistics of each model are shown below the table. PANSS: positive and negative syndrome scale; GAF: Global Assessment of Functioning

^a^ Overall Statistical Model: i) Main effects model F = 0.605 and Adj-R^2^ = 0.018, ii) Interaction model F = 2.397 and Adj-R^2^ = 0.074

^b^ Overall Statistical Model: i) Main effects model F = 0.414 and Adj-R^2^ = 0.027, ii) Interaction model F = 2.504 and Adj-R^2^ = 0.079

We also found a significant *NRN1*-rs4960155 x *CACNA1C*-rs1006737 epistasis effect on GAF scores. As indicated in Table 3, no main genotypic effects were detected, whereas their interaction was significant. When the interaction term was included, the model’s overall fit significantly improved (Δ-R^2^ = 0.112, p-value of the change = 0.002). As shown in Fig. 2B, patients presented inverse patterns in GAF scores as a function of both *NRN1* and *CACNA1C* genotypes. This effect did not substantially change when CPZ was added as a covariable in the model (β = 0.582, SE = 6.832, *p* = 0.003, model Adj-R^2^ = 0.102).

### Epistatic effects on SZ brain cortical measures

**Fig. 3:**
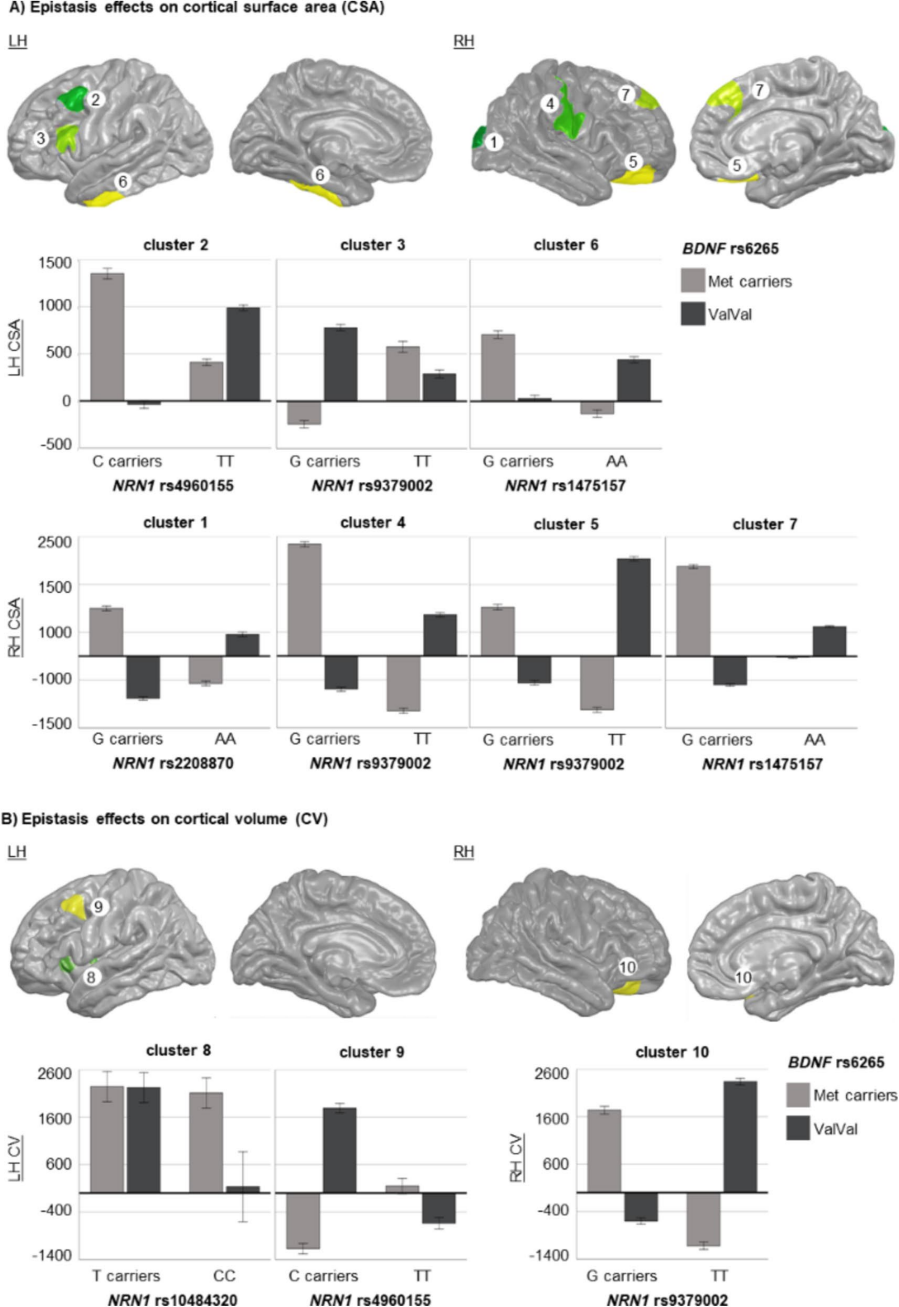
Brain regions where significant epistatic effects between *NRN1* and *BDNF* were detected in the whole-brain FreeSurfer analyses. We represented significant gene-gene interactions related to cortical surface area (CSA) (**A**) and cortical volume (CV) (**B**) on the pial surface, where a specific colour distinguishes each cluster, and both lateral and medial views are presented for each hemisphere (LH: left hemisphere; RH: right hemisphere). The bar plots depict the marginal mean scores (± 2 standard error) for each epistatic group

Regarding the interaction between *NRN1* SNPs and *BDNF*-rs6265, we detected many significant effects affecting the CSA of frontal, parietal and temporal regions. We also found that some epistasis effects regulated the CV of frontal regions (Table 4; Fig. 3).

**Table 4:** Significant clusters of interaction between *NRN1* and *BDNF* on different cortical measures (CM), such as cortical surface area (CSA), cortical thickness (CT) and cortical volume (CV)

Genetic variants	CM	CN	H	MNI coordinates			Z	CWP	Size	Label
Epistasis with *BDNF*										
*NRN1*-rs2208870 x *BDNF*-rs6265	CSA	1	R	22.4	-94.3	12.3	3.86	0.0002	796.61	Lateral occipital
*NRN1*-rs4960155 x *BDNF*-rs6265	CSA	2	L	-40.3	13.8	44.8	-5.36	0.0005	813.94	Caudal middle frontal
*NRN1-*rs9379002 x BDNF-rs6265	CSA	3	L	-52.3	21.6	15.0	4.64	0.0025	646.83	Parsopercularis
	CSA	4	R	52.6	-11.5	37.1	5.94	0.0001	969.25	Postcentral
	CSA	5	R	16.7	38.2	-17.9	5.07	0.0001	833.06	Lateral orbitofrontal
*NRN1*-rs1475157 x *BDNF*-rs6265	CSA	6	L	-46.9	-40.8	-15.4	4.34	0.0001	1284.28	Inferior temporal
	CSA	7	R	7.7	47.5	37.3	4.57	0.0033	342.39	Superior frontal
*NRN1-*rs10484320 x *BDNF*-rs6265	CV	8	L	-27.3	21.7	-4.1	-6.33	0.0013	529.04	Lateral orbitofrontal
*NRN1*-rs4960155 x *BDNF*-rs6265	CV	9	L	-40.3	13.0	40.1	-5.05	0.0001	756.48	Caudal middle frontal
*NRN1*-rs9379002 x *BDNF*-rs6265	CV	10	R	20.5	30.4	-13.6	4.56	0.0019	499.85	Lateral orbitofrontal
Epistasis with *CACNA1C*										
*NRN1-*rs1475157 x *CACNA1C*-rs1006737	CSA	11	L	-47.7	-12.0	-29.4	4.48	0.0025	771.82	Inferior temporal
*NRN1-*rs12333117 x *CACNA1C*-rs1006737	CV	12	L	-44.2	26.9	25.6	-3.67	0.0005	576.61	Rostral middle frontal

For each cluster are given per hemisphere (H: left (L) and right (R)), with the cluster number (CN), the main peak MNI coordinates, the Z value, the cluster-wise p-value (CWP), the size (as the number of vertices), and the label according to the Deskian-Killiany atlas included in FreeSurfer

**Fig. 4:**
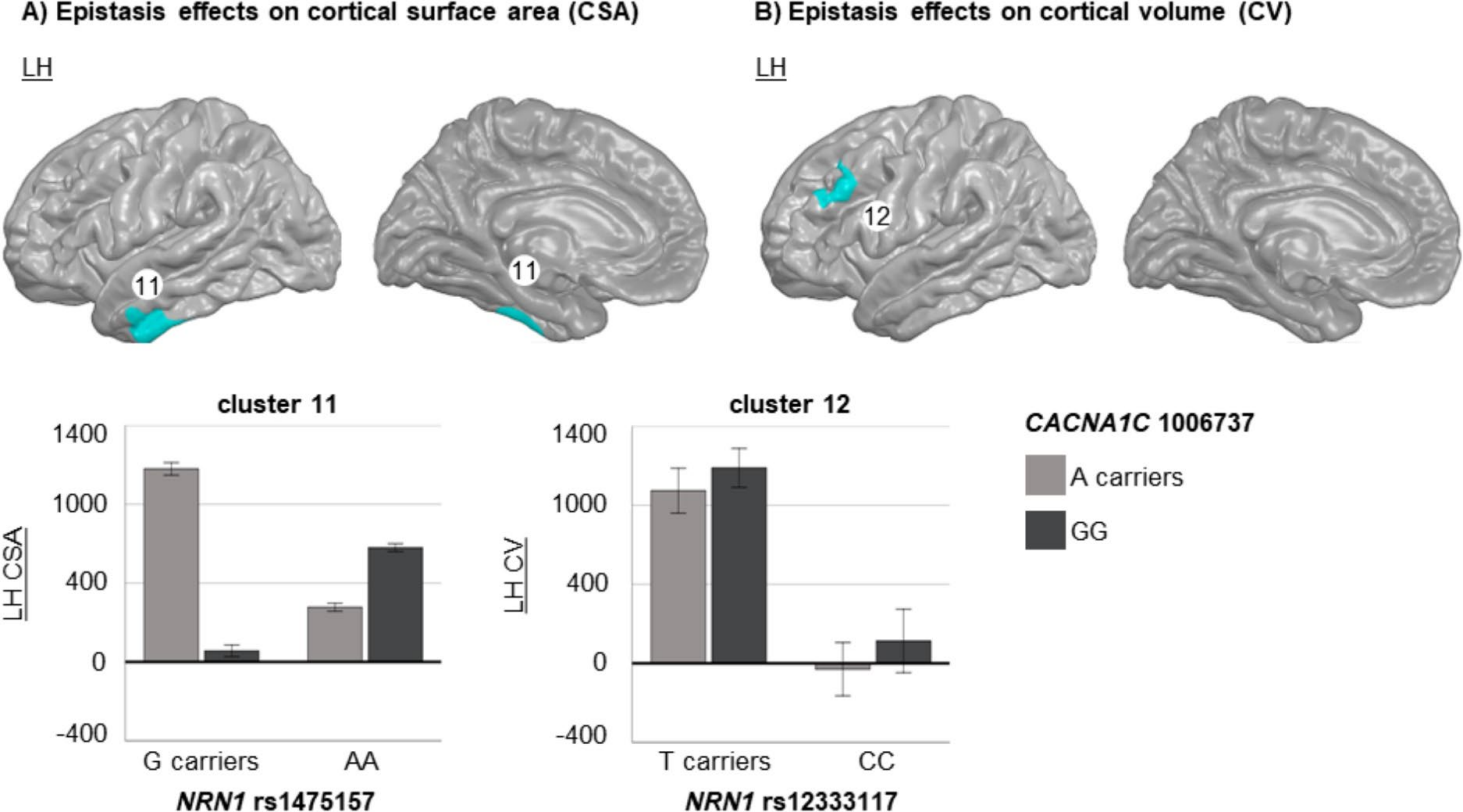
Brain regions where significant epistatic effects between *NRN1* and *CACNA1C* were detected in the whole-brain FreeSurfer analyses. We represented significant gene-gene interactions related to cortical surface area (CSA) (**A**) and cortical volume (CV) (**B**) on the pial surface, where each cluster is distinguished by a specific colour, and both lateral and medial views are presented for each hemisphere (LH: left hemisphere; RH: right hemisphere). The bar plots depict the marginal mean scores (± 2 standard error) for each epistatic group

Concerning the interaction between *NRN1* SNPs and *CACNA1C*-rs1006737, we detected significant effects modulating the CSA of temporal regions and the CV of frontal regions (Table 4; Fig. 4).

All the results remained significant after incorporating CPZ as a covariate in the model (data not shown).

### Cortical measures effects on clinical features

**Fig. 5:**
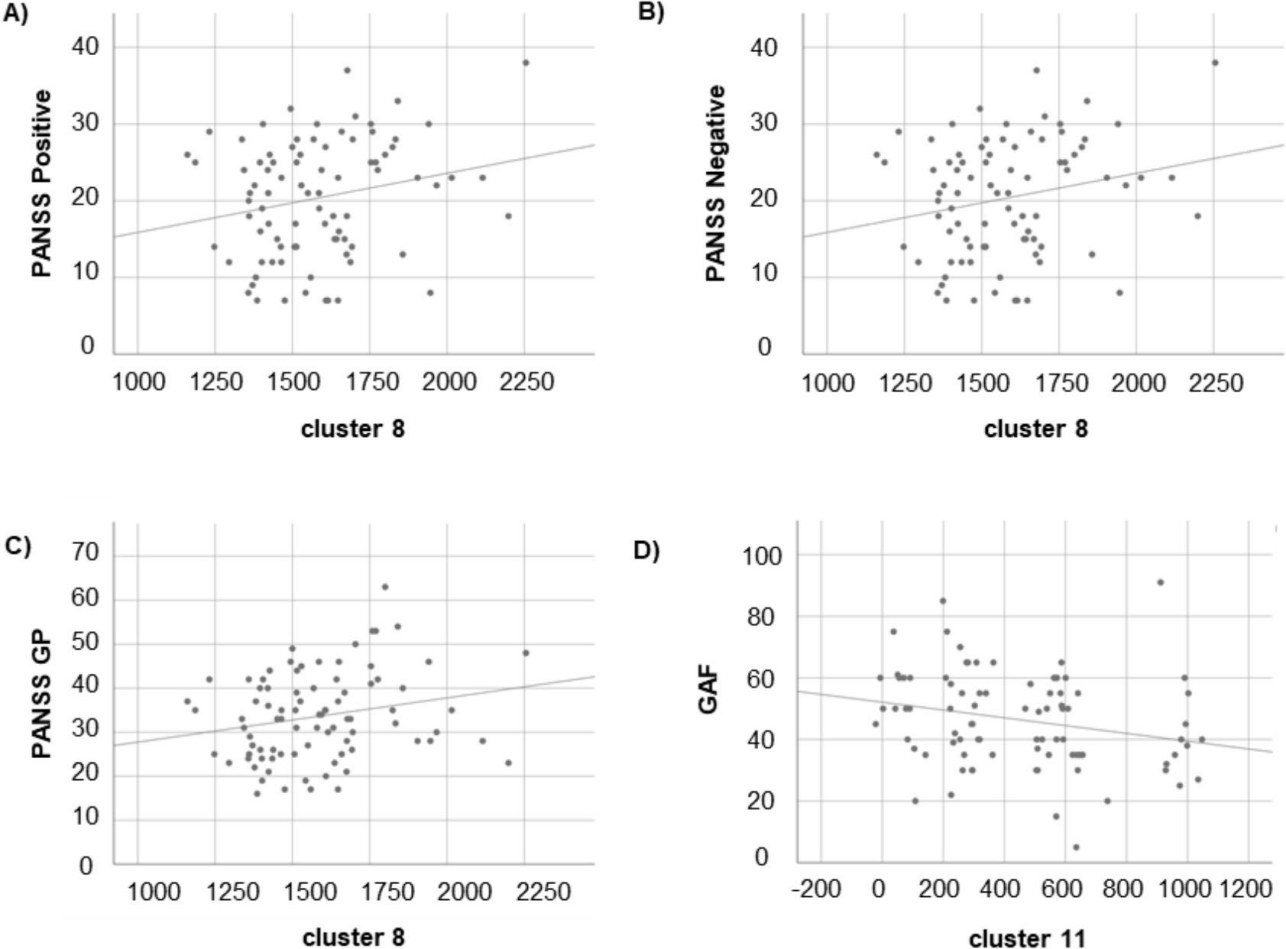
Scatter plots showing the significant correlations between brain clusters and clinical measures within subjects with SZ. For both clinical and morphometric measures, the reported values signify estimated marginal means. These means have been adjusted for potential confounding factors, including age and sex. In the case of brain measures, the adjustments also encompass intracranial volume. PANSS: positive and negative syndrome scale; GP: general psychopathology; GAF: Global Assessment Functioning; CV: cortical volume; CSA: cortical surface area; L-LOFC: left lateral orbitofrontal cortex

We found a significant association between the cluster comprising the left lateral orbitofrontal cortex (L-LOFC) (cluster 8) and PANSS subscales and total scores (Table 5). As illustrated in Fig. 5A, B and C, greater volumes of L-LOFC were associated with elevated PANSS scores, which are indicative of more severe symptoms. These effects ramined significant when CPZ was incorporated as a covariate in the model (Positive β = 0.322, SE = 0.003, *p* = 0.004, model Adj-R^2^ = 0.063; Negative β = 0.269, SE = 0.004, *p* = 0.014, model Adj-R^2^ = 0.105; General Psychopathology β = 0.281, SE = 0.005, *p* = 0.011, model Adj-R^2^ = 0.059; Total scores β = 0.340, SE = 0.009, *p* = 0.001, model Adj-R^2^ = 0.116). We also observed a significant correlation between the inferior temporal region (cluster 11) and GAF scores (Table 5). As depicted in Fig. 5D, greater volumes of the inferior temporal cortex were associated with lower GAF values, which are indicative of worse functioning. This effect did not change when CPZ was added as a covariate in the model (β = -0.243, SE = 0.006, p = 0.024, model Adj-R2 = 0.082).

**Table 5:** Linear regression models showing significant associations between brain clusters and clinical measures

Clinical outcomes	Cluster predictors			β	SE	Adj-R^2^	p-value
	CM	CN	Label				
PANSS Positive	CV	8	Lateral orbitofrontal	0.296	0.003	0.054	0.007
PANSS Negative	CV	8	Lateral orbitofrontal	0.227	0.004	0.091	0.038
PANSS General Psychopathology	CV	8	Lateral orbitofrontal	0.250	0.005	0.050	0.021
PANSS Total	CV	8	Lateral orbitofrontal	0.303	0.010	0.085	0.005
GAF	CSA	11	Inferior temporal	-0.269	0.006	0.031	0.021

The table shows the cortical measures (CM), such as cortical volume (CV) and cortical suface area (CSA), the cluster number (CN) and its location (label) according to the Desikan-Killian Atlas; the clinical outcomes (Positive and Negative syndrome scale (PANSS) and Global Assessment Functioning (GAF)); the statistical parameters of each regression model  (β, standardized regression coefficient; SE, standard error; Adj-R^2^, adjusted R^2^)

### Brain structure as a mediator of the epistatic effects on clinical features in SZ

**Fig. 6:**
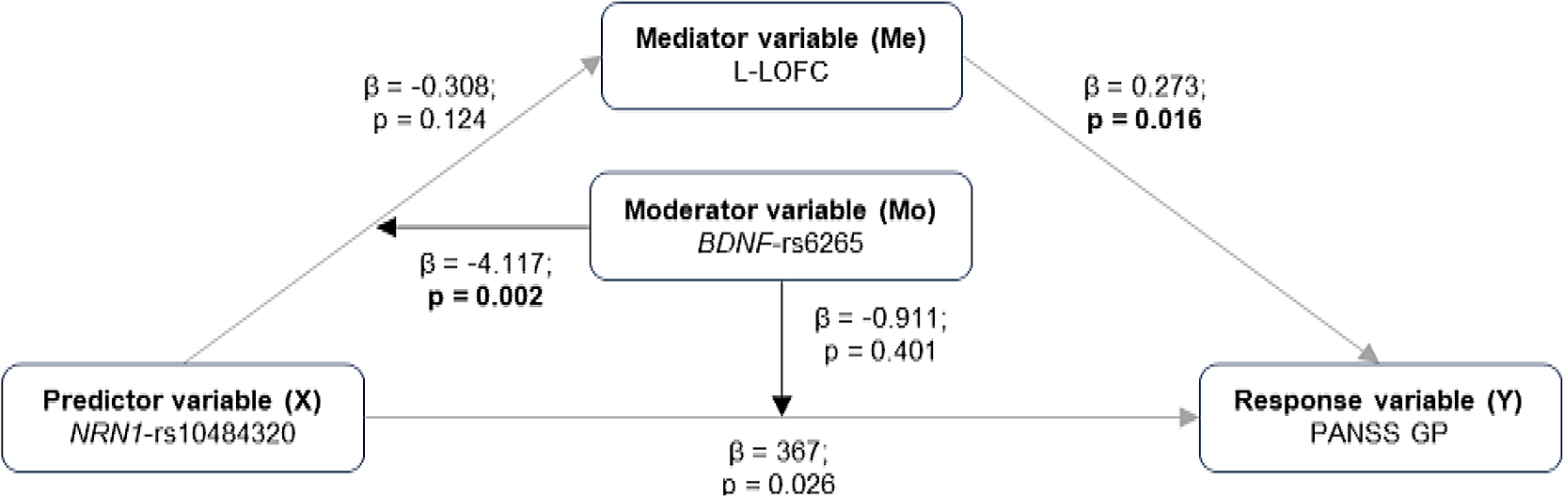
Conceptual diagram showing the significant moderated mediation model. Epistatic effects between *NRN1-*rs10484320 x *BDNF*-rs6265 on the left lateral orbitofrontal cortex (L-LOFC) volume mediates the epistatic effects on Positive and Negative Symptoms Scale (PANSS) General Psychopathology

Given the *NRN1-*rs10484320 x *BDNF*-rs6265 epistatic effect on both PANSS general psychopathology (Table 3; Fig. 2A) and the CV of the L-LOFC (Table 4; Fig. 3, cluster 8), we explored whether this brain cluster mediated the detected epistatic effect on psychopathology. As shown in Table 6 and schematised in Fig. 6, after extracting the mean values of the L-LOFC cluster for each individual, we confirmed that *NRN1-*rs10484320 x *BDNF*-rs6265 epistasis significantly accounted for variation of the L-LOFC volume. However, when the *NRN1-*rs10484320 x *BDNF*-rs6265 interaction and the L-LOFC volume were included in the same model to predict PANSS general psychopathology, the mediator was significantly associated, but the interaction did not retain its significance. The significant index of moderated mediation indicates that the influence of *NRN1*-rs10484320 x *BDNF*-rs6265 on PANSS general psychopathology is entirely mediated by its impact on L-LOFC volume. This effect remained significant when CPZ was added as a covariate in the model (IMM = -25.839; SE = 13.942; Bootstrap 95% CI [-59.225 – -5.108], *p* = 0.024)

**Table 6:** Moderated mediation effect of left lateral orbitofrontal cortex (L-LOFC, cluster 8) volume on the impact of *NRN1*-rs10484320 x *BDNF*-rs6265 epistasis on the Positive and Negative Symptoms Scale General Psychopathology (PANSS-GP)

Outcome variable	Models	Predictor variables	β / IMM*	SE	Bootstrap 95% CI	p-value
Mediator						
L-LOFC	a1	*NRN1*-rs10484320	-0.308	86.681	-295.80 – 21.14	0.124
	a2	*BDNF*-rs6265	-0.048	84.922	-175.477 – 118.905	0.812
	a3	*NRN1*-rs10484320 *x BDNF*-rs6265	-4.117	477.362	-2912.76 – -1070.41	0.002
Response variable						
PANSS PG	c’1	*NRN1*-rs10484320	0.367	2.980	1.429 – 12.595	0.026
	c’2	*BDNF*-rs6265	0.294	2.994	0.494 – 11.202	0.046
	c’3	*NRN1*-rs10484320 *x BDNF*-rs6265	-0.911	24.842	-68.574 – 51.022	0.401
	b1	L-LOFC	0.273	0.005	0.002 – 0.025	0.016
Moderated mediation						
*NRN1*-rs10484320 *x BDNF*-rs6265 → L-LOFC → PANSS PG			-24.24*	13.905	-59.132 – -3.467	0.036

The table shows the statistical parameters of each model  (β, standardized regression coefficient; SE, standard error; 95% confidence interval (CI); IMM, index of moderated mediation)

## Discussion

Based on data coming from several animal and cellular studies that highlight the molecular links between *NRN1, BDNF* and *CACNA1C* genes (Fujino et al. 2003; Wibrand et al. 2006), this study explores the neurobiological pathways through which the epistasis between these synaptic plasticity-related genes may be associated with the clinical presentation of SZ. By combining genetic, neuroimaging and clinical data, the main findings show the epistasis between *NRN1* and its interactors (*BDNF* and *CACNA1C)* on SZ clinical features and indicate that this joint genetic background may exert its effect through the impact on neuroanatomical measures.

Regarding our analyses exploring the epistatic effect on the risk for SZ, we did not detect any significant association. While the individual effect of *NRN1*, *BDNF* and *CACNA1C* on the risk for SZ is reported in different studies, there is little evidence showing that *NRN1* role seems to be not independent of *BDNF* (Fatjó-Vilas et al. 2016; Prats et al. 2017), and no previous study has explored the epistasis with *CACNA1C.* Thus, the counterpart to the novelty in our study is the challenge in comparability, as there is only one prior study (Fatjó-Vilas et al. 2016) that described a significant interaction effect between *NRN1*-rs9379002 and *BDNF*-rs6265 on a broader clinical spectrum, including both SZ and bipolar disorder. These discrepancies could be due to differences in diagnosis and sample size. Nonetheless, the current results do not indicate a direct epistatic effect on the risk of SZ, but reveal significant epistatic effects relevant to the clinical presentation of the disorder.

In this sense, we found a significant interaction between *NRN1*-rs10484320 and *BDNF*-rs6265 impacting PANSS general psychopathology, which encompasses symptoms like anxiety, guilt, tension, depression, and disorientation (Kay et al. 1987). Albeit earlier studies noted the negative impact of *BDNF*-rs6265 ValVal genotype on various PANSS subscales (Chang et al. 2009; Numata et al. 2006; Zhai et al. 2013), our data revealed that *NRN1*-rs10484320 modifies this effect. We observed more severe symptoms in patients carrying the *NRN1* T allele and the *BDNF* ValVal genotype or the *NRN1 *CC genotype and the *BDNF* Met allele than in those carrying the opposite genotypic combinations. We also identified a significant interaction between *NRN1*-rs4960155 and *CACNA1C*-rs1006737 affecting GAF scores, summarising personal, social, and psychological functioning (Endicott et al. 1976). Again, previous research has established the *CACNA1C*-rs1006737 A allele as a risk factor for SZ through GWAS and meta-analytic approaches (Liu et al. 2020; Trubetskoy et al. 2022) and has demonstrated its detrimental effect on longitudinal GAF scores and recovery after psychotic episodes (Heilbronner et al. 2015). Our data adds to such evidence by revealing that *NRN1*-rs4960155 modulates the *CACNA1C* effects. Patients with the *NRN1* TT genotype and the A allele for *CACNA1C* and those with the *NRN1* GG genotype but carrying the C allele for *CACNA1C* exhibited poorer functioning. It is worth mentioning that both *NRN1* SNPs, rs10484320 and rs4960155, have been previously associated with the risk of SZ-spectrum disorders and have been found to influence IQ scores among these individuals (Chandler et al. 2010; Fatjó-Vilas et al. 2016).

Nevertheless, taking into account the clinical heterogeneity of SZ and its complex aetiology, expecting a direct influence of genetic background on the disorder’s clinical presentation seems unrealistic; instead, genetic networks might modulate a lower-level trait, which, in turn, sustains the manifestation of symptoms (Glahn et al. 2010; Meijer et al. 2021). Therefore, following other previous studies (Kirschner et al. 2020; Miranda et al. 2019; Sudre et al. 2020), we have proposed that deconstructing SZ into biologically validated and stable trait markers, such as brain structural measures and investigating their role in mediating symptomatology could help to fill the gap in the path from synaptic plasticity genetic variability to the complex and heterogeneous clinical presentation of the disorder.

In this regard, we discovered that *NRN1*-rs2208870 and several upstream variants (rs10484320, rs4960155, rs9379002, rs1475157) interact with *BDNF*-rs6265 to modulate CSA and CV among individuals with SZ. Additionally, we identified that *NRN1*-rs1475157 and *NRN1*-12333117 interact with *CACNA1C*-rs1006737, modulating the CSA and CV, respectively, among individuals with SZ. These findings highlight the role of epistatic interactions involving *NRN1*, *BDNF*, and *CACNA1C* in contributing to the strong genetic underpinnings of CSA, which is estimated to have a heritability of 91% (Eyler et al. 2012). The critical roles of these genes in neural development (Sasi et al. 2017; Yao et al. 2018) also support its inclusion in the pool of genetic factors influencing CSA, which is driven by regulatory elements active during prenatal cortical development (Grasby et al. 2020).

Those brain regions significantly modulated by epistasis effects, mainly frontal regions as well as some parietal and temporal regions, have been previously reported to present CSA and CV reductions in patients with SZ (Erp et al. 2018; Madre et al. 2020; Rimol et al. 2012). Voxel-based morphometry meta-analyses have also evidenced CV reductions, especially in frontotemporal regions (Bora et al. 2011; Honea et al. 2005). Though our analysis was confined to patients with SZ patients, our findings indicate that these specific epistatic combinations distinctly impact brain structure in this group. Thus, it is plausible that these genetic interactions might also play a role in the molecular mechanisms contributing to the previously mentioned differences in cortical structure between patients and HS.

From all the epistasis effects on brain structure, we highlight the *NRN1-*rs10484320 x *BDNF*-rs6265 interaction effect on the L-LOFC volume since these genetic variants also jointly modulate PANSS general psychopathology. Our moderated mediation model was designed to deep into the relationship between these significant associations and confirmed the mediation effect of the L-LOFC in the relationship between the *NRN1-*rs10484320 x *BDNF*-rs6265 epistasis and the PANSS general psychopathology scores.

However, contrary to the expected, the same patients who presented the lowest PANSS general psychopathology scores, those carriers of the CC genotype for *NRN1-*rs10484320 and the ValVal genotype for *BDNF*-rs6265, were the ones with the smaller left L-LOFC volume. The L-LOFC, as part of a functional network including the medial prefrontal cortex (Öngür and Price 2000), plays multiple roles, such as the integration of multiple sensory information, modulation of visceral reactions, and participation in learning, prediction, and decision-making for emotional and reward-related behaviours (Kringelbach and Rolls 2004; Rolls 2004). Findings in SZ regarding structural alterations of the OFC are controversial. While some studies have reported volume reductions (Madre et al. 2020; Rimol et al. 2012), others have described increased volume of the left OFC (Lacerda et al. 2007). These incongruities are not clarified by exploring the relationship with symptoms, as both volume increase and decrease have been associated with SZ symptomatology (Baaré et al. 1999; Gur et al. 2000; Koutsouleris et al. 2008; Lacerda et al. 2007; Nakamura et al. 2008). Interestingly, proteomic studies have linked N-methyl-D-aspartate (NMDA) receptors hypofunction and disruption of calcium homeostasis with OFC volumetric alterations in SZ (Nascimento and Martins-de-Souza 2015; Velásquez et al. 2019). At the clinical level, decreasing glutamatergic neurotransmission through NMDA receptor antagonists generates and worsens psychotic traits, suggesting its significance as a major pathway for symptom development in SZ (Lewis and González-Burgos, 2007). In fact, *NRN1* expression in the cortex is regulated by Ca^2+^ signalling via the NMDA receptor (Fujino et al. 2003), and NMDA receptor-mediated neurotransmission and plasticity are particularly affected by *BDNF*-rs6265 genotype within the hippocampus and infralimbic medial prefrontal cortex (Ninan et al. 2010; Pattwell et al. 2012). A significant pathway in the development of positive and negative symptoms may result from the reduced activity of glutamate, mainly mediated through the NMDA receptor (Lewis and González-Burgos, 2007). Therefore, both neurotrophic factors might play a role in the molecular pathways underlying the volumetric differences of the L-LOFC in SZ subjects that are behaviourally reflected in general psychopathology. Thus, our findings offer insight into the previously debated results, providing evidence that these genetic factors may contribute to volumetric variability among individuals, which, in turn, may underlie the emergence of general psychopathology during lifespan.

To understand how those genetic variants modulate psychopathology through their impact on brain structure, the functional consequences of those polymorphisms must be considered. On the one hand, animal and cell-based models have demonstrated that the *BDNF*-rs6265 Met variant affects both its intracellular distribution and activity-dependent secretion (Chen et al. 2005, 2006; Chiaruttini et al. 2009; Egan et al. 2003). On the other hand, data from the GTEx Project identifies *BDNF*-rs6265 and *NRN1*-rs10484320 variants as expression quantitative trait loci (eQTLs) affecting the expression of both genes in the brain context but does not indicate an epistatic effect. However, it is essential to note that statistical interaction between polymorphisms does not necessarily imply a direct impact on gene expression; instead, the identified epistasis could reflect the influence of these polymorphisms on intermediate pathways.

Finally, we acknowledge several limitations in our study, with the primary concern being the sample size. To mitigate potential overfitting, we calculated the smallest detectable effect in our sample for both risk assessment and clinical associations and applied the Bonferroni threshold to select significant epistasis. Concerning power estimation related to brain structure analyses it is discouraged due to the inherent properties of whole-brain approaches. Additionally, power calculations using vertices inside a significant cluster may create circularity because these vertices have already been selected for having highly different values (Vul et al. 2009). Despite these challenges, we employed various methodological strategies to avoid type I errors. Firstly, we selected polymorphic variants with known associations with SZ and described functional interactions. Secondly, we applied the cluster-wise correction method to correct for multiple comparisons and selected only those clusters that met the Bonferroni criterion. Simultaneously, to enhance the robustness of our findings and avoid potential pitfalls, we connected epistatic effects on symptomatology and brain structure through a mediation exploration. These approaches allowed for the concurrent detection of the interaction effect between *NRN1*-rs10484320 and *BDNF*-rs6265 on SZ PANSS general psychopathology and L-LOFC volume while describing that this effect on the clinical manifestation is fully mediated by brain structure. However, we must acknowledge the potential for type II errors that may have impeded the detection of epistatic effects in other regions. Another limitation is that our study did not directly investigate the molecular mechanisms underlying the genetic interactions that contribute to the clinical presentation of SZ. It is important to note that our analyses focus on identifying statistical relationships, which do not necessarily imply direct biological connections. However, the biological plausibility of our findings is supported by prior cellular and animal models describing functional interactions related to synaptic plasticity between the studied genes. Nevertheless, further studies are necessary to validate the statistical and biological impact of genetic interactions on brain structure and symptoms. Lastly, while the homogeneity of our sample concerning ethnicity and demographic variables minimises potential errors, it also restricts the generalizability of our results. Upcoming research should strive for larger samples with more diverse representations to enhance the external validity of our findings.

## Conclu﻿sions

In conclusion, our study adds clinical significance to the well-described molecular relationship between *NRN1* and its molecular interactors, *BDNF* and *CACNA1C*, since we provide the first evidence of their epistatic impact on PANSS general psychopathology through its effect on L-LOFC volume specifically within subjects with SZ.

### Electronic supplementary material

Below is the link to the electronic supplementary material.

Supplementary Material 1

## Data Availability

No datasets were generated or analysed during the current study.
