# Supplementary material for: NRN1 epistasis with BDNF and CACNA1C: mediation effects on symptom severity through neuroanatomical changes in schizophrenia
Source: Brain Struct Funct. 2024 May 9;229(5):1299–315. doi: 10.1007/s00429-024-02793-5 (PMC11147852; doi:10.1007/s00429-024-02793-5)
Supplement: Supplementary file 1 — Supplementary Material 1 [file 429_2024_2793_MOESM1_ESM.docx]

**SUPPLEMENTARY MATERIALS**

**Submission to Brain Structure and Function**

*NRN1* EPISTASIS WITH *BDNF* AND *CACNA1C*: MEDIATION EFFECTS ON SYMPTOM SEVERITY THROUGH NEUROANATOMICAL CHANGES IN SCHIZOPHRENIA.

Carmen Almodóvar-Payá^1,2,3,#^; Maria Guardiola-Ripoll^1,#^; Maria Giralt-López^4,5^; Maitane Oscoz-Irurozqui^1,6^, Erick Jorge Canales-Rodríguez^1,3,7^, Mercè Madre^1,3,8^, Joan Soler-Vidal^1,3,9^, Núria Ramiro^10^, Luis Felipe Callado^3,11,12^, Bárbara Arias^2,3,13^; Carme Gallego^14^; Edith Pomarol-Clotet^1,3,+^; Mar Fatjó-Vilas^1,2,3,a,+^.

1. FIDMAG Germanes Hospitalàries Research Foundation, Barcelona, Spain.

2. Departament de Biologia Evolutiva, Ecologia i Ciències Ambientals, Facultat de Biologia, Universitat de Barcelona, Barcelona, Spain.

3. CIBERSAM (Biomedical Research Network in Mental Health; Instituto de Salud Carlos III), Madrid, Spain.

4. Department of Child and Adolescent Psychiatry, Germans Trias i Pujol University Hospital (HUGTP), Barcelona, Spain.

5. Department of Psychiatry and Legal Medicine, Faculty of Medicine, Autonomous University of Barcelona (UAB), Barcelona, Spain.

6. Red de Salud Mental de Gipuzkoa, Osakidetza-Basque Health Service, Gipuzkoa, Spain.

7. Signal Processing Laboratory (LTS5), École Polytechnique Fédérale de Lausanne (EPFL), Lausanne, Switzerland

8. Mental Health, IR SANT PAU, Hospital de la Santa Creu i Sant Pau, Universitat Autònoma Barcelona, Barcelona, Spain

9. Hospital Benito Menni, Germanes Hospitalàries, Sant Boi de Llobregat, Barcelona, Spain.

10. Hospital San Rafael, Germanes Hospitalàries, Barcelona, Spain.

11. Department of Pharmacology, University of the Basque Country (UPV/EHU), Bizkaia, Spain.

12. BioBizkaia Health Research Institute, Bizkaia, Spain.

13. Institut de Biomedicina de la Universitat de Barcelona (IBUB), Barcelona, Spain.

14. Department of Cells and Tissues, Molecular Biology Institute of Barcelona (IBMB-CSIC), Barcelona, Spain.

^#^ Co-first authors

+ Co-senior authors.

^a^ Corresponding author: M Fatjó-Vilas, FIDMAG Germanes Hospitalàries Research Foundation. Av Jordà 8, 08035 Barcelona, Spain. E-mail address: mfatjo-vilas@fidmag.org


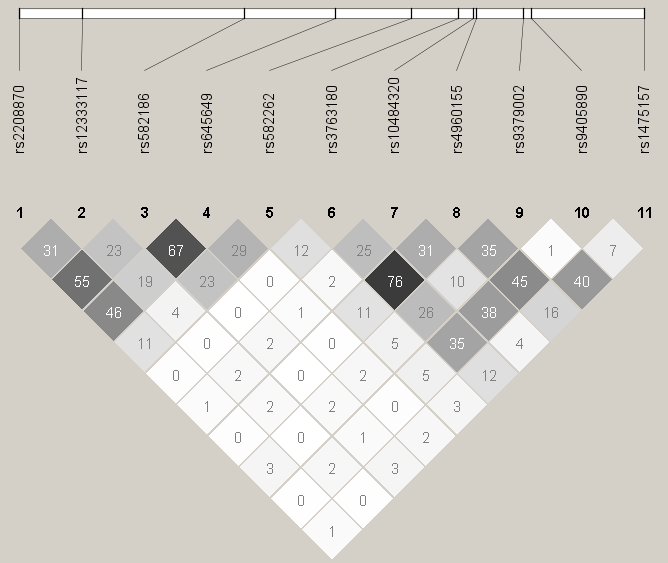
Figure 1. Linkage disequilibrium (LD) plot generated by Haploview 4.2 software based on pairwise r^2^ for the SNPs across Neuritin-1 (NRN1) gene. The linkage is represented in a colour gradient where r^2^ = 0 is given in white, 0 < r^2^ < 1 is given in shades of grey and r^2^ = 1 is given in black.

| Table S1. Epistatic interaction between Neuritin-1 *NRN1* single nucleotide polymorphisms (SNPs) and Brain Derived Neurotrophic Factor (*BDNF)* polymorphism rs6265. The table includes dichotomized genotypes for each of the epistatic pairs and its corresponding count (frequency) and the p-value of the logistic regression (adjusted by sex). | | | | | |
| --- | --- | --- | --- | --- | --- |
| *NRN1* SNPs | | *BDNF* rs6265 | Healthy Subjects | Schizophrenia | p-value |
| rs2208870 | GGorGA | Met carriers | 15 (0.17) | 21 (0.24) | 0.925 |
|  |  | ValVal | 35 (0.41) | 31 (0.35) |  |
|  | AA | Met carriers | 14 (0.16) | 18 (0.2) |  |
|  |  | ValVal | 22 (0.26) | 19 (0.21) |  |
| rs12333117 | TTorTC | Met carriers | 20 (0.24) | 27 (0.3) | 0540 |
|  |  | ValVal | 29 (0.34) | 31 (0.35) |  |
|  | CC | Met carriers | 9 (0.11) | 12 (0.13) |  |
|  |  | ValVal | 27 (0.32) | 19 (0.21) |  |
| rs582186 | AAorAG | Met carriers | 13 (0.15) | 23 (0.26) | 0.386 |
|  |  | ValVal | 40 (0.47) | 35 (0.4) |  |
|  | GG | Met carriers | 16 (0.19) | 16 (0.18) |  |
|  |  | ValVal | 16 (0.19) | 14 (0.16) |  |
| rs645649 | CCorCG | Met carriers | 16 (0.19) | 24 (0.27) | 0.694 |
|  |  | ValVal | 41 (0.48) | 36 (0.4) |  |
|  | GG | Met carriers | 13 (0.15) | 15 (0.17) |  |
|  |  | ValVal | 16 (0.19) | 14 (0.16) |  |
| rs582262 | CCorCG | Met carriers | 13 (0.15) | 13 (0.15) | 0.462 |
|  |  | ValVal | 31 (0.36) | 28 (0.32) |  |
|  | GG | Met carriers | 16 (0.19) | 24 (0.28) |  |
|  |  | ValVal | 26 (0.3) | 22 (0.25) |  |
| rs3763180 | TTorTG | Met carriers | 24 (0.28) | 22 (0.25) | 0.624 |
|  |  | ValVal | 43 (0.51) | 28 (0.31) |  |
|  | GG | Met carriers | 5 (0.06) | 17 (0.19) |  |
|  |  | ValVal | 13 (0.15) | 22 (0.25) |  |
| rs10484320 | TTorTC | Met carriers | 5 (0.06) | 18 (0.2) | 0.118 |
|  |  | ValVal | 23 (0.27) | 24 (0.27) |  |
|  | CC | Met carriers | 24 (0.28) | 21 (0.24) |  |
|  |  | ValVal | 34 (0.4) | 26 (0.29) |  |
| rs4960155 | CCorCT | Met carriers | 23 (0.29) | 28 (0.31) | 0.846 |
|  |  | ValVal | 43 (0.54) | 31 (0.35) |  |
|  | TT | Met carriers | 4 (0.05) | 11 (0.12) |  |
|  |  | ValVal | 10 (0.13) | 19 (0.21) |  |
| rs9379002 | GGorGT | Met carriers | 14 (0.16) | 17 (0.19) | 0.591 |
|  |  | ValVal | 20 (0.24) | 25 (0.28) |  |
|  | TT | Met carriers | 15 (0.18) | 21 (0.24) |  |
|  |  | ValVal | 36 (0.42) | 25 (0.28) |  |
| rs9405890 | CCorCT | Met carriers | 10 (0.12) | 23 (0.26) | 0.228 |
|  |  | ValVal | 31 (0.36) | 30 (0.34) |  |
|  | TT | Met carriers | 19 (0.22) | 16 (0.18) |  |
|  |  | ValVal | 26 (0.3) | 20 (0.22) |  |
| rs1475157 | GGorGA | Met carriers | 9 (0.13) | 10 (0.09) | 0.073 |
|  |  | ValVal | 9 (0.13) | 17 (0.16) |  |
|  | AA | Met carriers | 20 (0.29) | 47 (0.44) |  |
|  |  | ValVal | 30 (0.44) | 33 (0.31) |  |

| Table S2. Epistatic interaction between Neuritin-1 (*NRN1)* single nucleotide polymorphisms (SNPs) and calcium voltage-gated channel subunit alpha1 C (*CACNA1C)* polymorphism rs1006737. The table includes dichotomized genotypes for each of the epistatic pairs and its corresponding count (frequency), the p-value and the odds ratio (OR) of the logistic regression (adjusted by sex). | | | | | |
| --- | --- | --- | --- | --- | --- |
| *NRN1* SNPs | | *CACNA1C* rs1006737 | Healthy Subjects | Schizophrenia | p-value (OR ^a^) |
| rs2208870 | GGorGA | AAorAG | 22 (0.26) | 26 (0.29) | 0.405 |
|  |  | GG | 28 (0.33) | 26 (0.29) |  |
|  | AA | AAorAG | 18 (0.21) | 16 (0.18) |  |
|  |  | GG | 18 (0.21) | 21 (0.24) |  |
| rs12333117 | TTorTC | AAorAG | 22 (0.26) | 25 (0.28) | 0.671 |
|  |  | GG | 27 (0.32) | 33 (0.37) |  |
|  | CC | AAorAG | 18 (0.21) | 17 (0.19) |  |
|  |  | GG | 18 (0.21) | 14 (0.16) |  |
| rs582186 | AAorAG | AAorAG | 25 (0.29) | 27 (0.31) | 0.979 |
|  |  | GG | 28 (0.33) | 31 (0.35) |  |
|  | GG | AAorAG | 15 (0.18) | 14 (0.16) |  |
|  |  | GG | 17 (0.2) | 16 (0.18) |  |
| rs645649 | CCorCG | AAorAG | 27 (0.31) | 28 (0.31) | 0.796 |
|  |  | GG | 30 (0.35) | 32 (0.36) |  |
|  | GG | AAorAG | 13 (0.15) | 14 (0.16) |  |
|  |  | GG | 16 (0.19) | 15 (0.17) |  |
| rs582262 | CCorCG | AAorAG | 18 (0.21) | 20 (0.23) | 0.413 |
|  |  | GG | 26 (0.3) | 21 (0.24) |  |
|  | GG | AAorAG | 22 (0.26) | 22 (0.25) |  |
|  |  | GG | 20 (0.23) | 24 (0.28) |  |
| rs3763180 | TTorTG | AAorAG | 28 (0.33) | 26 (0.29) | 0.037 (4.34) ^b^ |
|  |  | GG | 39 (0.46) | 24 (0.27) |  |
|  | GG | AAorAG | 12 (0.14) | 16 (0.18) |  |
|  |  | GG | 6 (0.07) | 23 (0.26) |  |
| rs10484320 | TTorTC | AAorAG | 14 (0.16) | 16 (0.18) | 0.151 |
|  |  | GG | 14 (0.16) | 26 (0.29) |  |
|  | CC | AAorAG | 26 (0.3) | 26 (0.29) |  |
|  |  | GG | 32 (0.37) | 21 (0.24) |  |
| rs4960155 | CCorCT | AAorAG | 29 (0.36) | 32 (0.36) | 0.028 (5.44) ^b^ |
|  |  | GG | 37 (0.46) | 27 (0.3) |  |
|  | TT | AAorAG | 9 (0.11) | 10 (0.11) |  |
|  |  | GG | 5 (0.06) | 20 (0.22) |  |
| rs9379002 | GGorGT | AAorAG | 17 (0.2) | 21 (0.24) | 0.971 |
|  |  | GG | 17 (0.2) | 21 (0.24) |  |
|  | TT | AAorAG | 23 (0.27) | 21 (0.24) |  |
|  |  | GG | 28 (0.33) | 25 (0.28) |  |
| rs9405890 | CCorCT | AAorAG | 19 (0.22) | 27 (0.3) | 0.528 |
|  |  | GG | 22 (0.26) | 26 (0.29) |  |
|  | TT | AAorAG | 21 (0.24) | 15 (0.17) |  |
|  |  | GG | 24 (0.28) | 21 (0.24) |  |
| rs1475157 | GGorGA | AAorAG | 10 (0.12) | 12 (0.13) | 0.593 |
|  |  | GG | 9 (0.1) | 14 (0.16) |  |
|  | AA | AAorAG | 30 (0.35) | 30 (0.34) |  |
|  |  | GG | 37 (0.43) | 33 (0.37) |  |
| ^a^ OR is given when the p-value < 0.05. | | | | | |
| ^b^ Not significant after Bonferroni correction. | | | | | |

| Table S3. Epistatic interaction between Neuritin-1 *NRN1* single nucleotide polymorphisms (SNPs) and Brain Derived Neurotrophic Factor (*BDNF)* polymorphism rs6265 or calcium voltage-gated channel subunit alpha1 C (*CACNA1C)* polymorphism rs1006737 on positive sub-scale of the Positive and Negative Syndrome Scale (PANSS). The table includes dichotomized genotypes for each of the epistatic pairs and the corresponding raw mean (standard deviation) and the p-value of the interaction term of the linear regression (adjusted by sex, age, and chlorpromazine equivalents). | | | | | | | |
| --- | --- | --- | --- | --- | --- | --- | --- |
| *NRN1* SNPs | | *BDNF* rs6265 | PANSS Positive | p-value | *CACNA1C* rs1006737 | PANSS Positive | p-value |
| rs2208870 | GGorGA | Met carriers | 15.19 (5.56) | 0.278 | AAorAG | 15.04 (5.5) | 0.892 |
|  |  | ValVal | 17.03 (4.95) |  | GG | 17.54 (4.72) |  |
|  | AA | Met carriers | 19.22 (5.75) |  | AAorAG | 17.63 (4.87) |  |
|  |  | ValVal | 18.42 (7.51) |  | GG | 19.71 (7.7) |  |
| rs12333117 | TTorTC | Met carriers | 16.48 (6.23) | 0.955 | AAorAG | 15 (5.28) | 0.889 |
|  |  | ValVal | 16.77 (6.6) |  | GG | 17.88 (6.91) |  |
|  | CC | Met carriers | 18.33 (5.21) |  | AAorAG | 17.53 (5.27) |  |
|  |  | ValVal | 18.84 (4.8) |  | GG | 20 (4.15) |  |
| rs582186 | AAorAG | Met carriers | 16.35 (6.2) | 0.137 | AAorAG | 15.48 (5.63) | 0.254 |
|  |  | ValVal | 18.17 (5.85) |  | GG | 19.16 (5.87) |  |
|  | GG | Met carriers | 18.06 (5.56) |  | AAorAG | 16.79 (4.98) |  |
|  |  | ValVal | 15.86 (6.5) |  | GG | 17.25 (6.95) |  |
| rs645649 | CCorCG | Met carriers | 16.25 (6.08) | 0.060 | AAorAG | 15.86 (5.87) | 0.498 |
|  |  | ValVal | 18.39 (5.91) |  | GG | 19 (5.85) |  |
|  | GG | Met carriers | 18.33 (5.64) |  | AAorAG | 16.36 (4.34) |  |
|  |  | ValVal | 15.43 (5.96) |  | GG | 17.47 (7.14) |  |
| rs582262 | CCorCG | Met carriers | 18.15 (6.8) | 0.989 | AAorAG | 17.15 (6.2) | 0.885 |
|  |  | ValVal | 18.36 (6.79) |  | GG | 19.38 (7.14) |  |
|  | GG | Met carriers | 16.25 (5.57) |  | AAorAG | 15 (4.36) |  |
|  |  | ValVal | 16.55 (4.82) |  | GG | 17.67 (5.59) |  |
| rs3763180 | TTorTG | Met carriers | 16.91 (5.83) | 0.836 | AAorAG | 15.73 (5.76) | 0.807 |
|  |  | ValVal | 17.14 (6.67) |  | GG | 18.46 (6.57) |  |
|  | GG | Met carriers | 17.24 (6.23) |  | AAorAG | 16.5 (4.79) |  |
|  |  | ValVal | 18.09 (5.17) |  | GG | 18.57 (6.05) |  |
| rs10484320 | TTorTC | Met carriers | 16.72 (6.29) | 0.072 | AAorAG | 17.69 (5.16) | 0.387 |
|  |  | ValVal | 19.63 (5.72) |  | GG | 18.81 (6.64) |  |
|  | CC | Met carriers | 17.33 (5.74) |  | AAorAG | 15 (5.32) |  |
|  |  | ValVal | 15.65 (5.73) |  | GG | 18.14 (5.88) |  |
| rs4960155 | CCorCT | Met carriers | 16.86 (6.07) | 0.986 | AAorAG | 15.5 (5.53) | 0.265 |
|  |  | ValVal | 17.23 (6.37) |  | GG | 18.89 (6.49) |  |
|  | TT | Met carriers | 17.55 (5.8) |  | AAorAG | 17.7 (4.64) |  |
|  |  | ValVal | 18.11 (5.52) |  | GG | 18 (6.03) |  |
| rs9379002 | GGorGT | Met carriers | 17.41 (6.26) | 0.894 | AAorAG | 15.48 (4.73) | 0.070 |
|  |  | ValVal | 17.96 (5.35) |  | GG | 20 (5.72) |  |
|  | TT | Met carriers | 16.33 (5.55) |  | AAorAG | 16.57 (6) |  |
|  |  | ValVal | 17.16 (6.7) |  | GG | 16.96 (6.39) |  |
| rs9405890 | CCorCT | Met carriers | 16.17 (6.5) | 0.053 | AAorAG | 16 (4.88) | 0.490 |
|  |  | ValVal | 18.7 (5.53) |  | GG | 19.27 (6.75) |  |
|  | TT | Met carriers | 18.31 (4.92) |  | AAorAG | 16.07 (6.33) |  |
|  |  | ValVal | 15.85 (6.44) |  | GG | 17.57 (5.59) |  |
| rs1475157 | GGorGA | Met carriers | 18.33 (5.85) | 0.750 | AAorAG | 16.58 (5.85) | 0.753 |
|  |  | ValVal | 18.12 (6.86) |  | GG | 19.57 (6.76) |  |
|  | AA | Met carriers | 16.67 (6) |  | AAorAG | 15.8 (5.24) |  |
|  |  | ValVal | 17.27 (5.62) |  | GG | 18.06 (6.08) |  |

| Table S4. Epistatic interaction between Neuritin-1 *NRN1* single nucleotide polymorphisms (SNPs) and Brain Derived Neurotrophic Factor (*BDNF)* polymorphism rs6265 or calcium voltage-gated channel subunit alpha1 C (*CACNA1C)* polymorphism rs1006737 on negative sub-scale of the Positive and Negative Syndrome Scale (PANSS). The table includes dichotomized genotypes for each of the epistatic pairs and the corresponding raw mean (standard deviation) and the p-value of the interaction term of the linear regression (adjusted by sex, age, and chlorpromazine equivalents). | | | | | | | |
| --- | --- | --- | --- | --- | --- | --- | --- |
| *NRN1* SNPs | | *BDNF* rs6265 | PANSS Negative | p-value | *CACNA1C r*s1006737 | PANSS Negative | p-value |
| rs2208870 | GGorGA | Met carriers | 19.29 (5.98) | 0.082 | AAorAG | 19.73 (7.65) | 0.457 |
|  |  | ValVal | 20.1 (8.36) |  | GG | 19.81 (7.36) |  |
|  | AA | Met carriers | 23.5 (7.24) |  | AAorAG | 23 (8.85) |  |
|  |  | ValVal | 19.53 (8.41) |  | GG | 20.29 (7.31) |  |
| rs12333117 | TTorTC | Met carriers | 22.33 (6.64) | 0.259 | AAorAG | 20.96 (9.44) | 0.745 |
|  |  | ValVal | 19.19 (9.33) |  | GG | 20.42 (7.42) |  |
|  | CC | Met carriers | 18.75 (6.89) |  | AAorAG | 21 (6.15) |  |
|  |  | ValVal | 21 (6.32) |  | GG | 19.07 (7.04) |  |
| rs582186 | AAorAG | Met carriers | 19.57 (6.21) | 0.107 | AAorAG | 19.93 (7.62) | 0.925 |
|  |  | ValVal | 19.97 (8.23) |  | GG | 19.71 (7.41) |  |
|  | GG | Met carriers | 23.63 (7.17) |  | AAorAG | 22.21 (9.03) |  |
|  |  | ValVal | 18.79 (8.33) |  | GG | 20.63 (7.16) |  |
| rs645649 | CCorCG | Met carriers | 19.17 (6.38) | 0.047 ^a^ | AAorAG | 20.21 (7.63) | 0.813 |
|  |  | ValVal | 20.19 (8.22) |  | GG | 19.41 (7.49) |  |
|  | GG | Met carriers | 24.53 (6.4) |  | AAorAG | 22.5 (9.29) |  |
|  |  | ValVal | 19.07 (8.73) |  | GG | 21.33 (6.81) |  |
| rs582262 | CCorCG | Met carriers | 19.77 (6.13) | 0.332 | AAorAG | 20.4 (8.62) | 0.927 |
|  |  | ValVal | 19.93 (8.21) |  | GG | 19.38 (6.51) |  |
|  | GG | Met carriers | 21.79 (7.44) |  | AAorAG | 21.5 (7.93) |  |
|  |  | ValVal | 19.82 (8.6) |  | GG | 20.25 (8.17) |  |
| rs3763180 | TTorTG | Met carriers | 20.32 (6.24) | 0.679 | AAorAG | 19.15 (7.78) | 0.419 |
|  |  | ValVal | 18.14 (8.22) |  | GG | 19.04 (7.18) |  |
|  | GG | Met carriers | 22.41 (7.58) |  | AAorAG | 23.94 (8.18) |  |
|  |  | ValVal | 22.09 (8.04) |  | GG | 21.04 (7.36) |  |
| rs10484320 | TTorTC | Met carriers | 21.61 (7.68) | 0.378 | AAorAG | 22.81 (8.33) | 0.630 |
|  |  | ValVal | 21.71 (8.11) |  | GG | 20.96 (7.59) |  |
|  | CC | Met carriers | 20.9 (6.2) |  | AAorAG | 19.85 (8.04) |  |
|  |  | ValVal | 18.19 (8.26) |  | GG | 18.86 (6.84) |  |
| rs4960155 | CCorCT | Met carriers | 21.14 (6) | 0.424 | AAorAG | 19.59 (7.79) | 0.125 |
|  |  | ValVal | 18.42 (8.46) |  | GG | 19.85 (7.19) |  |
|  | TT | Met carriers | 21.45 (8.97) |  | AAorAG | 25.4 (8.19) |  |
|  |  | ValVal | 22.26 (7.64) |  | GG | 20.25 (7.53) |  |
| rs9379002 | GGorGT | Met carriers | 20.71 (7.7) | 0.334 | AAorAG | 22.38 (8.21) | 0.433 |
|  |  | ValVal | 21.36 (8.09) |  | GG | 19.81 (7.44) |  |
|  | TT | Met carriers | 22.14 (5.93) |  | AAorAG | 19.57 (8.1) |  |
|  |  | ValVal | 18.4 (8.39) |  | GG | 20.56 (7.15) |  |
| rs9405890 | CCorCT | Met carriers | 21 (7.35) | 0.235 | AAorAG | 22.3 (7.82) | 0.207 |
|  |  | ValVal | 21.13 (8.37) |  | GG | 19.81 (7.86) |  |
|  | TT | Met carriers | 21.56 (6.25) |  | AAorAG | 18.6 (8.53) |  |
|  |  | ValVal | 18 (8.02) |  | GG | 20.29 (6.62) |  |
| rs1475157 | GGorGA | Met carriers | 20.56 (5.85) | 0.506 | AAorAG | 21.08 (8.38) | 0.432 |
|  |  | ValVal | 21.76 (8.78) |  | GG | 21.57 (7.55) |  |
|  | AA | Met carriers | 21.43 (7.19) |  | AAorAG | 20.93 (8.24) |  |
|  |  | ValVal | 18.91 (8) |  | GG | 19.36 (7.15) |  |
| ^a^ Not significant after Bonferroni correction. | | | | | | | |

| Table S5. Epistatic interaction between Neuritin-1 *NRN1* single nucleotide polymorphisms (SNPs) and Brain Derived Neurotrophic Factor (*BDNF)* polymorphism rs6265 or calcium voltage-gated channel subunit alpha1 C (*CACNA1C)* polymorphism rs1006737 on general psychopathology (GP) sub-scale of the Positive and Negative Syndrome Scale (PANSS). The table includes dichotomized genotypes for each of the epistatic pairs and the corresponding raw mean (standard deviation) and the p-value of the interaction term of the linear regression (adjusted by sex, age, and chlorpromazine equivalents). | | | | | | | |
| --- | --- | --- | --- | --- | --- | --- | --- |
| *NRN1* SNPs | | *BDNF* rs6265 | PANSS GP | p-value | *CACNA1C* rs1006737 | PANSS GP | p-value |
| rs2208870 | GGorGA | Met carriers | 32.67 (8.2) | 0.522 | AAorAG | 33.69 (10.02) | 0.524 |
|  |  | ValVal | 32.84 (9.99) |  | GG | 31.85 (8.45) |  |
|  | AA | Met carriers | 35.61 (9.77) |  | AAorAG | 34.31 (9.16) |  |
|  |  | ValVal | 33.79 (11.37) |  | GG | 34.95 (11.66) |  |
| rs12333117 | TTorTC | Met carriers | 34 (9.15) | 0.811 | AAorAG | 32.84 (9.65) | 0.541 |
|  |  | ValVal | 32.32 (10.57) |  | GG | 33.3 (10.21) |  |
|  | CC | Met carriers | 34.08 (8.91) |  | AAorAG | 35.53 (9.56) |  |
|  |  | ValVal | 34.63 (10.31) |  | GG | 33.07 (9.93) |  |
| rs582186 | AAorAG | Met carriers | 32.52 (8.02) | 0.039 ^a^ | AAorAG | 33.56 (9.6) | 0.567 |
|  |  | ValVal | 34.66 (11.22) |  | GG | 34.03 (10.58) |  |
|  | GG | Met carriers | 36.19 (10.03) |  | AAorAG | 34.57 (10.24) |  |
|  |  | ValVal | 29.43 (7.72) |  | GG | 31.69 (8.94) |  |
| rs645649 | CCorCG | Met carriers | 32.71 (7.9) | 0.050 ^a^ | AAorAG | 33.5 (9.43) | 0.470 |
|  |  | ValVal | 34.58 (11.06) |  | GG | 34.13 (10.42) |  |
|  | GG | Met carriers | 36.13 (10.38) |  | AAorAG | 34.79 (10.21) |  |
|  |  | ValVal | 29.64 (7.84) |  | GG | 31.33 (9.14) |  |
| rs582262 | CCorCG | Met carriers | 31.77 (7.9) | 0.079 | AAorAG | 33.35 (9.83) | 0.530 |
|  |  | ValVal | 34.54 (11.23) |  | GG | 33.95 (10.91) |  |
|  | GG | Met carriers | 35 (9.73) |  | AAorAG | 34.45 (9.57) |  |
|  |  | ValVal | 31.5 (9.29) |  | GG | 32.29 (9.68) |  |
| rs3763180 | TTorTG | Met carriers | 34.05 (8.41) | 0.635 | AAorAG | 32.08 (9.14) | 0.154 |
|  |  | ValVal | 32.07 (10.87) |  | GG | 33.88 (10.62) |  |
|  | GG | Met carriers | 34 (9.89) |  | AAorAG | 36.94 (9.83) |  |
|  |  | ValVal | 34.64 (9.91) |  | GG | 32.57 (9.53) |  |
| rs10484320 | TTorTC | Met carriers | 31.06 (8.58) | 0.003 ^b^ | AAorAG | 36 (8.04) | 0.376 |
|  |  | ValVal | 36.71 (10.86) |  | GG | 33.23 (11.4) |  |
|  | CC | Met carriers | 36.57 (8.68) |  | AAorAG | 32.65 (10.37) |  |
|  |  | ValVal | 29.96 (9.05) |  | GG | 33.24 (8.27) |  |
| rs4960155 | CCorCT | Met carriers | 33.61 (8.08) | 0.914 | AAorAG | 31.84 (8.52) | 0.018 ^a^ |
|  |  | ValVal | 32.13 (10.36) |  | GG | 34 (10.18) |  |
|  | TT | Met carriers | 35.09 (11.27) |  | AAorAG | 40.6 (10.21) |  |
|  |  | ValVal | 34.95 (10.59) |  | GG | 32.2 (9.95) |  |
| rs9379002 | GGorGT | Met carriers | 35.24 (10.78) | 0.404 | AAorAG | 34.67 (9.83) | 0.653 |
|  |  | ValVal | 32.84 (9.37) |  | GG | 32.95 (10.15) |  |
|  | TT | Met carriers | 33.67 (7.05) |  | AAorAG | 33.19 (9.53) |  |
|  |  | ValVal | 33.56 (11.58) |  | GG | 33.96 (9.98) |  |
| rs9405890 | CCorCT | Met carriers | 31.35 (9.02) | 0.009 ^a^ | AAorAG | 34.74 (7.42) | 0.271 |
|  |  | ValVal | 35 (9.86) |  | GG | 32.04 (11.4) |  |
|  | TT | Met carriers | 37.88 (7.57) |  | AAorAG | 32.47 (12.78) |  |
|  |  | ValVal | 30.5 (10.93) |  | GG | 34.71 (8) |  |
| rs1475157 | GGorGA | Met carriers | 36.22 (11.53) | 0.214 | AAorAG | 33.25 (11.95) | 0.705 |
|  |  | ValVal | 31.94 (10.8) |  | GG | 33.57 (10.62) |  |
|  | AA | Met carriers | 33.37 (8.16) |  | AAorAG | 34.2 (8.7) |  |
|  |  | ValVal | 33.85 (10.34) |  | GG | 33.09 (9.91) |  |
| ^a^ Not significant after Bonferroni correction. | | | | | | | |
| ^b^ Significant after Bonferroni correction, reported on the manuscript. | | | | | | | |

| Table S6. Epistatic interaction between Neuritin-1 *NRN1* single nucleotide polymorphisms (SNPs) and Brain Derived Neurotrophic Factor (*BDNF)* polymorphism rs6265 or calcium voltage-gated channel subunit alpha1 C (*CACNA1C)* polymorphism rs1006737 on the Positive and Negative Syndrome Scale (PANSS) total scores. The table includes dichotomized genotypes for each of the epistatic pairs and the corresponding raw mean (standard deviation) and the p-value of the interaction term of the linear regression (adjusted by sex, age, and chlorpromazine equivalents). | | | | | | | |
| --- | --- | --- | --- | --- | --- | --- | --- |
| *NRN1* SNPs | | *BDNF* rs6265 | PANSS Total | p-value | *CACNA1C* rs1006737 | PANSS Total | p-value |
| rs2208870 | GGorGA | Met carriers | 67.14 (14.56) | 0.185 | AAorAG | 68.46 (18.9) | 0.993 |
|  |  | ValVal | 69.97 (19.61) |  | GG | 69.19 (16.66) |  |
|  | AA | Met carriers | 78.33 (18.61) |  | AAorAG | 74.94 (20.85) |  |
|  |  | ValVal | 71.74 (24.75) |  | GG | 74.95 (23.24) |  |
| rs12333117 | TTorTC | Met carriers | 72.81 (17.76) | 0.569 | AAorAG | 68.8 (20.38) | 0.637 |
|  |  | ValVal | 68.29 (22.54) |  | GG | 71.61 (20.66) |  |
|  | CC | Met carriers | 71.17 (16.84) |  | AAorAG | 74.06 (18.74) |  |
|  |  | ValVal | 74.47 (19.59) |  | GG | 72.14 (18.52) |  |
| rs582186 | AAorAG | Met carriers | 68.43 (15.92) | 0.035 ^a^ | AAorAG | 68.96 (18.73) | 0.507 |
|  |  | ValVal | 72.8 (21.78) |  | GG | 72.9 (20.52) |  |
|  | GG | Met carriers | 77.88 (18.12) |  | AAorAG | 73.57 (21.99) |  |
|  |  | ValVal | 64.07 (20.44) |  | GG | 69.56 (18.93) |  |
| rs645649 | CCorCG | Met carriers | 68.13 (15.65) | 0.020 ^a^ | AAorAG | 69.57 (18.66) | 0.636 |
|  |  | ValVal | 73.17 (21.58) |  | GG | 72.53 (20.3) |  |
|  | GG | Met carriers | 79 (18.17) |  | AAorAG | 73.64 (22.03) |  |
|  |  | ValVal | 64.14 (20.53) |  | GG | 70.13 (19.45) |  |
| rs582262 | CCorCG | Met carriers | 69.69 (16.33) | 0.214 | AAorAG | 70.9 (21.02) | 0.760 |
|  |  | ValVal | 72.82 (22.74) |  | GG | 72.71 (20.99) |  |
|  | GG | Met carriers | 73.04 (18.41) |  | AAorAG | 70.95 (18.86) |  |
|  |  | ValVal | 67.86 (19.93) |  | GG | 70.21 (19.75) |  |
| rs3763180 | TTorTG | Met carriers | 71.27 (16.11) | 0.646 | AAorAG | 66.96 (19.29) | 0.273 |
|  |  | ValVal | 67.36 (22.79) |  | GG | 71.38 (20.97) |  |
|  | GG | Met carriers | 73.65 (19.1) |  | AAorAG | 77.38 (19.12) |  |
|  |  | ValVal | 74.82 (19.39) |  | GG | 72.17 (19.08) |  |
| rs10484320 | TTorTC | Met carriers | 69.39 (18.15) | 0.017 ^a^ | AAorAG | 76.5 (17.85) | 0.373 |
|  |  | ValVal | 78.04 (21.14) |  | GG | 73 (21.68) |  |
|  | CC | Met carriers | 74.81 (16.52) |  | AAorAG | 67.5 (20.28) |  |
|  |  | ValVal | 63.81 (19.79) |  | GG | 70.24 (17.74) |  |
| rs4960155 | CCorCT | Met carriers | 71.61 (15.71) | 0.715 | AAorAG | 66.94 (17.98) | 0.035 ^a^ |
|  |  | ValVal | 67.77 (21.96) |  | GG | 72.74 (20.41) |  |
|  | TT | Met carriers | 74.09 (21.52) |  | AAorAG | 83.7 (20.28) |  |
|  |  | ValVal | 75.32 (20.37) |  | GG | 70.45 (19.52) |  |
| rs9379002 | GGorGT | Met carriers | 73.35 (20.24) | 0.933 | AAorAG | 72.52 (19.55) | 0.992 |
|  |  | ValVal | 72.16 (19.21) |  | GG | 72.76 (19.73) |  |
|  | TT | Met carriers | 72.14 (15.11) |  | AAorAG | 69.33 (20.14) |  |
|  |  | ValVal | 69.12 (23.83) |  | GG | 71.48 (20.55) |  |
| rs9405890 | CCorCT | Met carriers | 68.52 (17.71) | 0.018 ^a^ | AAorAG | 73.04 (15.85) | 0.409 |
|  |  | ValVal | 74.83 (19.54) |  | GG | 71.12 (21.83) |  |
|  | TT | Met carriers | 77.75 (15.57) |  | AAorAG | 67.13 (25.35) |  |
|  |  | ValVal | 64.35 (23.18) |  | GG | 72.57 (17.59) |  |
| rs1475157 | GGorGA | Met carriers | 75.11 (19.98) | 0.646 | AAorAG | 70.92 (23.98) | 0.558 |
|  |  | ValVal | 71.82 (24.03) |  | GG | 74.71 (21.62) |  |
|  | AA | Met carriers | 71.47 (16.67) |  | AAorAG | 70.93 (18.14) |  |
|  |  | ValVal | 70.03 (20.41) |  | GG | 70.52 (19.26) |  |
| ^a^ Not significant after Bonferroni correction. | | | | | | | |

| Table S7. Epistatic interaction between Neuritin-1 *NRN1* single nucleotide polymorphisms (SNPs) and Brain Derived Neurotrophic Factor (*BDNF)* polymorphism rs6265 or calcium voltage-gated channel subunit alpha1 C (*CACNA1C)* polymorphism rs1006737 on Global Assessment of Functioning scale (GAF). The table includes dichotomized genotypes for each of the epistatic pairs and the corresponding raw mean (standard deviation) and the p-value of the interaction term of the linear regression (adjusted by sex, age, and chlorpromazine equivalents). | | | | | | | |
| --- | --- | --- | --- | --- | --- | --- | --- |
| *NRN1* SNPs | | *BDNF* rs6265 | GAF | p-value | *CACNA1C* rs1006737 | GAF | p-value |
| rs2208870 | GGorGA | Met carriers | 45.67 (12.12) | 0.325 | AAorAG | 46.31 (16.04) | 0.185 |
|  |  | ValVal | 47.84 (15.87) |  | GG | 47.62 (12.8) |  |
|  | AA | Met carriers | 40.83 (15.19) |  | AAorAG | 49.75 (16.48) |  |
|  |  | ValVal | 49.79 (16.45) |  | GG | 42.14 (15.71) |  |
| rs12333117 | TTorTC | Met carriers | 41.26 (13.45) | 0.411 | AAorAG | 47.6 (17.71) | 0.218 |
|  |  | ValVal | 47.84 (17.62) |  | GG | 42.64 (14.53) |  |
|  | CC | Met carriers | 48.33 (13.37) |  | AAorAG | 47.65 (13.91) |  |
|  |  | ValVal | 49.79 (13.16) |  | GG | 51.14 (12.11) |  |
| rs582186 | AAorAG | Met carriers | 44.83 (12.72) | 0.664 | AAorAG | 47 (15.65) | 0.238 |
|  |  | ValVal | 48.8 (14.72) |  | GG | 47.42 (12.62) |  |
|  | GG | Met carriers | 41.44 (15.09) |  | AAorAG | 48.64 (18.02) |  |
|  |  | ValVal | 47.93 (19.79) |  | GG | 40.81 (16.61) |  |
| rs645649 | CCorCG | Met carriers | 44.63 (12.48) | 0.455 | AAorAG | 46.29 (15.82) | 0.139 |
|  |  | ValVal | 48.19 (14.96) |  | GG | 47.19 (12.49) |  |
|  | GG | Met carriers | 41.53 (15.62) |  | AAorAG | 50.29 (16.91) |  |
|  |  | ValVal | 49.57 (18.85) |  | GG | 40.87 (17.19) |  |
| rs582262 | CCorCG | Met carriers | 44 (13.64) | 0.321 | AAorAG | 46.1 (16.28) | 0.614 |
|  |  | ValVal | 46.39 (15.86) |  | GG | 45.19 (14.19) |  |
|  | GG | Met carriers | 43 (14.43) |  | AAorAG | 49 (16.18) |  |
|  |  | ValVal | 51.36 (16) |  | GG | 45.17 (15.18) |  |
| rs3763180 | TTorTG | Met carriers | 44.55 (12.35) | 0.521 | AAorAG | 50.73 (17.32) | 0.023 ^a^ |
|  |  | ValVal | 48 (18.13) |  | GG | 41.88 (12.77) |  |
|  | GG | Met carriers | 42 (15.45) |  | AAorAG | 42.56 (12.81) |  |
|  |  | ValVal | 49.32 (13.04) |  | GG | 48.61 (15.22) |  |
| rs10484320 | TTorTC | Met carriers | 42.33 (14.05) | 0.662 | AAorAG | 42 (11.24) | 0.122 |
|  |  | ValVal | 45.83 (13.66) |  | GG | 45.77 (15.15) |  |
|  | CC | Met carriers | 44.38 (13.58) |  | AAorAG | 51.08 (17.79) |  |
|  |  | ValVal | 51.12 (17.7) |  | GG | 44.43 (13.46) |  |
| rs4960155 | CCorCT | Met carriers | 43.11 (14.61) | 0.750 | AAorAG | 49.88 (16.32) | 0.002 ^b^ |
|  |  | ValVal | 47.39 (17.52) |  | GG | 40 (14.62) |  |
|  | TT | Met carriers | 44.27 (11.44) |  | AAorAG | 40.4 (13.69) |  |
|  |  | ValVal | 50.53 (13.23) |  | GG | 52.15 (10.57) |  |
| rs9379002 | GGorGT | Met carriers | 45.29 (14.64) | 0.652 | AAorAG | 49 (16.59) | 0.541 |
|  |  | ValVal | 51.48 (14.36) |  | GG | 48.95 (12.78) |  |
|  | TT | Met carriers | 42.33 (13.23) |  | AAorAG | 46.24 (15.88) |  |
|  |  | ValVal | 45.68 (17.2) |  | GG | 42.4 (15.16) |  |
| rs9405890 | CCorCT | Met carriers | 44.78 (15) | 0.228 | AAorAG | 47.04 (13.24) | 0.967 |
|  |  | ValVal | 46.53 (12.8) |  | GG | 44.46 (14.28) |  |
|  | TT | Met carriers | 41.5 (11.64) |  | AAorAG | 48.67 (20.78) |  |
|  |  | ValVal | 51.65 (19.74) |  | GG | 46.05 (14.58) |  |
| rs1475157 | GGorGA | Met carriers | 40.22 (12.67) | 0.273 | AAorAG | 43.17 (18.38) | 0.024 ^a^ |
|  |  | ValVal | 51.41 (16.66) |  | GG | 51.29 (13.35) |  |
|  | AA | Met carriers | 44.4 (14) |  | AAorAG | 49.4 (15.06) |  |
|  |  | ValVal | 47.12 (15.64) |  | GG | 42.58 (14.05) |  |
| ^a^ Not significant after Bonferroni correction. | | | | | | | |
| ^b^ Significant after Bonferroni correction, reported on the manuscript. | | | | | | | |

| Table S8. Epistatic interaction between Neuritin-1 *NRN1* single nucleotide polymorphisms (SNPs) and Brain Derived Neurotrophic Factor (*BDNF)* polymorphism rs6265 or calcium voltage-gated channel subunit alpha1 C (*CACNA1C)* polymorphism rs1006737 on Clinical Global Impression scale (CGI). The table includes dichotomized genotypes for each of the epistatic pairs and the corresponding raw mean (standard deviation) and the p-value of the interaction term of the linear regression (adjusted by sex, age, and chlorpromazine equivalents). | | | | | | | |
| --- | --- | --- | --- | --- | --- | --- | --- |
| *NRN1* SNPs | | *BDNF* rs6265 | CGI | p-value | *CACNA1C* rs1006737 | CGI | p-value |
| rs2208870 | GGorGA | Met carriers | 4.19 (0.81) | 0.133 | AAorAG | 4.62 (1.06) | 0.697 |
|  |  | ValVal | 4.74 (1.03) |  | GG | 4.42 (0.9) |  |
|  | AA | Met carriers | 4.67 (1.14) |  | AAorAG | 4.56 (1.21) |  |
|  |  | ValVal | 4.47 (1.17) |  | GG | 4.57 (1.12) |  |
| rs12333117 | TTorTC | Met carriers | 4.52 (0.98) | 0.454 | AAorAG | 4.56 (1.16) | 0.205 |
|  |  | ValVal | 4.68 (1.11) |  | GG | 4.64 (0.96) |  |
|  | CC | Met carriers | 4.17 (1.03) |  | AAorAG | 4.65 (1.06) |  |
|  |  | ValVal | 4.58 (1.07) |  | GG | 4.14 (1.03) |  |
| rs582186 | AAorAG | Met carriers | 4.17 (0.89) | 0.055 | AAorAG | 4.63 (1.04) | 0.594 |
|  |  | ValVal | 4.74 (1.04) |  | GG | 4.42 (0.99) |  |
|  | GG | Met carriers | 4.75 (1.06) |  | AAorAG | 4.5 (1.29) |  |
|  |  | ValVal | 4.36 (1.22) |  | GG | 4.63 (1.02) |  |
| rs645649 | CCorCG | Met carriers | 4.17 (0.87) | 0.038 ^a^ | AAorAG | 4.64 (1.03) | 0.506 |
|  |  | ValVal | 4.75 (1.02) |  | GG | 4.41 (0.98) |  |
|  | GG | Met carriers | 4.8 (1.08) |  | AAorAG | 4.5 (1.29) |  |
|  |  | ValVal | 4.36 (1.22) |  | GG | 4.67 (1.05) |  |
| rs582262 | CCorCG | Met carriers | 4.23 (0.93) | 0.269 | AAorAG | 4.75 (1.07) | 0.479 |
|  |  | ValVal | 4.79 (1.1) |  | GG | 4.48 (1.08) |  |
|  | GG | Met carriers | 4.54 (1.02) |  | AAorAG | 4.45 (1.14) |  |
|  |  | ValVal | 4.45 (1.06) |  | GG | 4.54 (0.93) |  |
| rs3763180 | TTorTG | Met carriers | 4.32 (0.99) | 0.403 | AAorAG | 4.54 (1.21) | 0.453 |
|  |  | ValVal | 4.75 (1.17) |  | GG | 4.58 (1.02) |  |
|  | GG | Met carriers | 4.53 (1.01) |  | AAorAG | 4.69 (0.95) |  |
|  |  | ValVal | 4.5 (0.96) |  | GG | 4.39 (0.99) |  |
| rs10484320 | TTorTC | Met carriers | 4.56 (1.1) | 0.897 | AAorAG | 4.94 (1) | 0.331 |
|  |  | ValVal | 4.79 (0.93) |  | GG | 4.54 (0.99) |  |
|  | CC | Met carriers | 4.29 (0.9) |  | AAorAG | 4.38 (1.13) |  |
|  |  | ValVal | 4.5 (1.21) |  | GG | 4.43 (1.03) |  |
| rs4960155 | CCorCT | Met carriers | 4.39 (0.99) | 0.408 | AAorAG | 4.56 (1.13) | 0.283 |
|  |  | ValVal | 4.81 (1.14) |  | GG | 4.67 (1.04) |  |
|  | TT | Met carriers | 4.45 (1.04) |  | AAorAG | 4.7 (1.06) |  |
|  |  | ValVal | 4.37 (0.96) |  | GG | 4.25 (0.91) |  |
| rs9379002 | GGorGT | Met carriers | 4.41 (1.12) | 0.718 | AAorAG | 4.33 (1.11) | 0.098 |
|  |  | ValVal | 4.52 (1.05) |  | GG | 4.62 (1.02) |  |
|  | TT | Met carriers | 4.38 (0.92) |  | AAorAG | 4.86 (1.06) |  |
|  |  | ValVal | 4.76 (1.13) |  | GG | 4.36 (0.99) |  |
| rs9405890 | CCorCT | Met carriers | 4.39 (1.12) | 0.468 | AAorAG | 4.56 (1.15) | 0.458 |
|  |  | ValVal | 4.73 (1.01) |  | GG | 4.62 (0.98) |  |
|  | TT | Met carriers | 4.44 (0.81) |  | AAorAG | 4.67 (1.05) |  |
|  |  | ValVal | 4.5 (1.19) |  | GG | 4.33 (1.02) |  |
| rs1475157 | GGorGA | Met carriers | 4.56 (0.88) | 0.604 | AAorAG | 4.58 (0.67) | 0.932 |
|  |  | ValVal | 4.53 (1.07) |  | GG | 4.5 (1.22) |  |
|  | AA | Met carriers | 4.37 (1.03) |  | AAorAG | 4.6 (1.25) |  |
|  |  | ValVal | 4.7 (1.1) |  | GG | 4.48 (0.91) |  |
| ^a^ Not significant after Bonferroni correction. | | | | | | | |
